# Fast nanopore sequencing data analysis with SLOW5

**DOI:** 10.1038/s41587-021-01147-4

**Published:** 2022-01-03

**Authors:** Hasindu Gamaarachchi, Hiruna Samarakoon, Sasha P. Jenner, James M. Ferguson, Timothy G. Amos, Jillian M. Hammond, Hassaan Saadat, Martin A. Smith, Sri Parameswaran, Ira W. Deveson

**Affiliations:** 1 Kinghorn Centre for Clinical Genomics, Garvan Institute of Medical Research, Sydney, New South Wales Australia; 2 School of Computer Science and Engineering, University of New South Wales, Sydney, New South Wales Australia; 3 CHU Sainte-Justine Research Centre, Montreal, Quebec Canada; 4 Department of Biochemistry and Molecular Medicine, Faculty of Medicine, University of Montreal, Montreal, Quebec Canada; 5 St Vincent’s Clinical School, Faculty of Medicine, University of New South Wales, Sydney, New South Wales Australia

**Keywords:** DNA sequencing, Genetics research

## Abstract

Nanopore sequencing depends on the FAST5 file format, which does not allow efficient parallel analysis. Here we introduce SLOW5, an alternative format engineered for efficient parallelization and acceleration of nanopore data analysis. Using the example of DNA methylation profiling of a human genome, analysis runtime is reduced from more than two weeks to approximately 10.5 h on a typical high-performance computer. SLOW5 is approximately 25% smaller than FAST5 and delivers consistent improvements on different computer architectures.

## Abstract (web summary)

Nanopore sequencing data are rapidly analyzed with parallel data access.

## Main

The emergence of nanopore sequencing is reshaping the landscape of genomics. Devices from Oxford Nanopore Technologies (ONT) enable sequencing of native DNA and RNA molecules with no theoretical upper limit on read length^1^. This supports the accurate assembly and phasing of repetitive genomes and metagenomes^2–6^; enhanced resolution of structural variation^7–11^ and spliced RNA transcripts^12^; and profiling of epigenetic and RNA modifications^13–18^. High-throughput ONT instruments (GridION and PromethION) have recently enabled cost-effective sequencing of large eukaryotic genomes^7,8,19^. However, large data volumes and computational bottlenecks have become a major impediment.

**Fig. 1:**
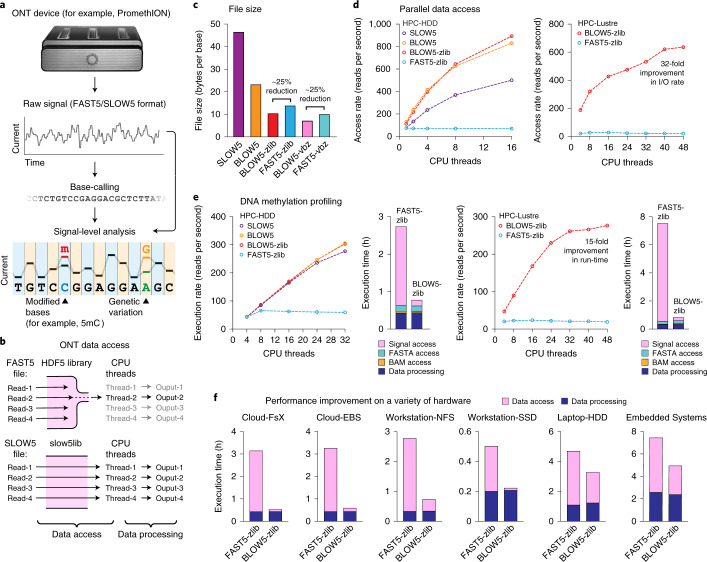
SLOW5 format enables efficient parallel analysis of nanopore signal data. **a**, Schematic diagram illustrating the typical life cycle of nanopore data. Raw current signal data are generated on an ONT sequencing device and written in FAST5 format. Raw data are base-called into sequence reads (FASTQ/FASTA format). Downstream analysis involving both base-called reads and raw signal data is used to identify genetic variants, epigenetic modifications (for example, 5mC) and other features. **b**, Schematic diagram illustrating the bottleneck in ONT signal data analysis. FAST5 file reading requires the HDF5 software library, which serializes file access requests by multiple CPU threads, preventing efficient parallel analysis. SLOW5 files are not dependent on the HDF5 library and are amenable to efficient parallel analysis. A more detailed mechanistic diagram is provided in Extended Data Fig. 1e. **c**, Bar chart shows the relative file sizes (bytes per base) of a typical human genome sequencing dataset in ASCII SLOW5 (purple), binary BLOW5 format with no compression (orange), zlib compression (red) and vbz compression (pink), compared to FAST5 format with zlib compression (blue) and vbz compression (teal). **d**, Dot plots show the rate of file access (reads per second) for the above file types, as a function of CPU threads used on two HPC systems: HPC-HDD (left) or HPC-Lustre (right). **e**, Dot plots show the rate of execution (reads per second) for DNA methylation calling for the same file types on HPC-HDD (left) and HPC-Lustre (right). For the instance of maximum CPU threads, bar charts show the time consumed by individual workflow components: FAST5/SLOW5 data access (pink), FASTA data access (teal), BAM data access (orange) and data processing (navy). **f**, Bar charts show the time consumed by data access (pink) and data processing (navy) during DNA methylation calling on a range of different computer systems. Full specifications are provided in Supplementary Table 2. Source data

ONT devices measure the displacement of ionic current as a DNA or RNA strand passes through a biological nanopore, recording time series signal data in FAST5 format (Fig. 1a and Supplementary Note 1). These data are translated, or ‘base-called’, into sequence reads (FASTQ format) before downstream analysis. Many bioinformatics tools also directly access the signal data to improve the accuracy of assembled genomes or detect fine signal perturbations that are indicative of DNA/RNA modifications, genetic variants or other features (Fig. 1a)^5,14,16–18^. However, nanopore signal data are large (~1.3-TB FAST5 files for ~30× human genome; Supplementary Table 1), and both base-calling and downstream analysis steps are computationally expensive.

**Extended Data Fig. 1:**
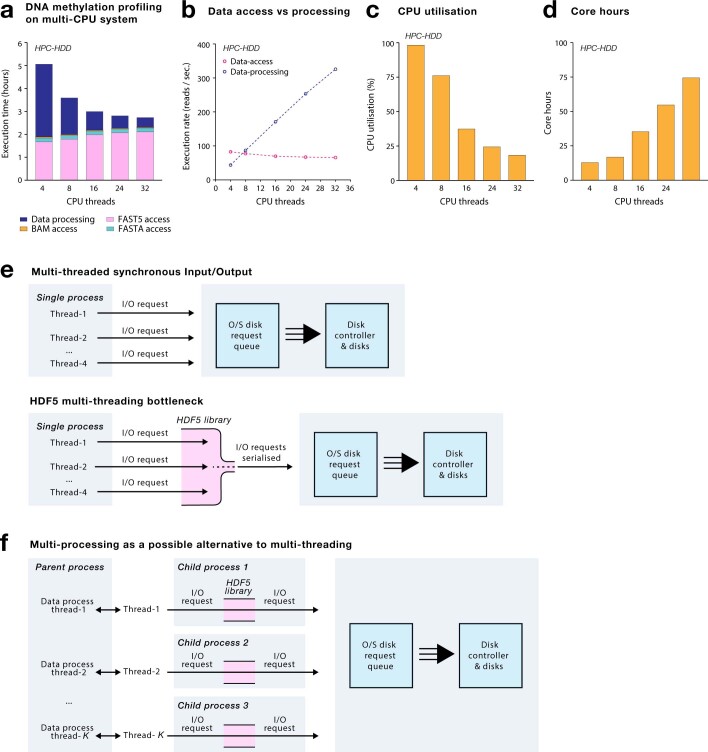
Inefficient parallel access is a major bottleneck in analysis of FAST5 files. **(a)** Bar chart shows the time consumed by individual components of a Nanopolish DNA methylation calling job with signal data input in FAST5 format: FAST5 data access (pink), FASTA data access (teal), BAM data access (orange) and data processing (navy). To assess the impact of multi-threading, the analysis was run with various numbers of CPU threads on the HPC-HDD system (see Supplementary Table 2). The analysis was run on a downsampled human genome sequencing dataset of 500,000 reads (see Supplementary Table 1). **(b)** Dot plots show the rate of file access and processing (reads / second) during the DNA methylation calling job above, as a function of CPU threads used. (c,d) Bar charts show the proportional CPU utilisation **(c)** and total core hours **(d)** during the DNA methylation calling jobs above. The definition of core-hours is provided in the Methods section. **(e)** The upper schematic illustrates the architecture of a job with multi-threaded synchronous file access (I/O). The lower schematic illustrates the bottleneck created by the HDF5 library that is required to read FAST5 files. The HDF5 library serialises I/O requests, making multi-threaded analysis highly inefficient and causing the observed decline in CPU utilisation with increasing numbers of CPU threads. **(f)** Schematic illustrates the architecture of a multi-processing approach that was implemented to circumvent this limitation in the HDF5 library. The multi-processing approach is viable but requires challenging software engineering and is not a generalisable, long-term solution.

**Extended Data Fig. 2:**
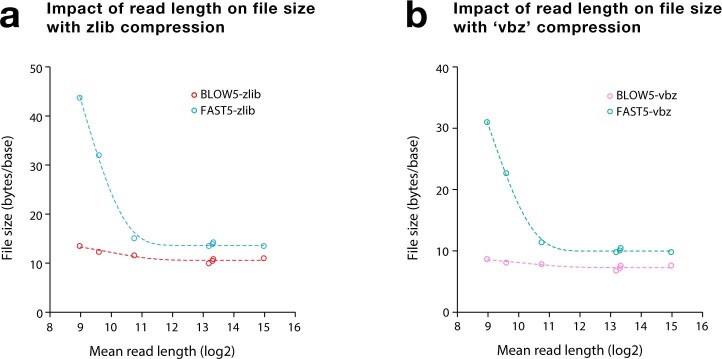
Impact of read length on file sizes for FAST5 vs BLOW5 files. (a,b) Dot plot show relative file sizes (bytes / base) of various datasets (see Supplementary Table 3) as a function of mean read length (shown on a log2 scale). File sizes are shown separately for FAST5-zlib vs BLOW5-zlib **(a)** and FAST5-vbz vs BLOW5-vbz **(b)** formats. File sizes are highly variable among different FAST5 files and largely stable among BLOW5 files. Libraries that have the shortest read lengths exhibit the largest space-savings, regardless of compression type.

Currently, the most popular signal-level analysis is DNA methylation profiling with the software Nanopolish/f5c^17,20^. We selected this example use case as the basis for an analysis of FAST5 data analysis on high-performance computing (HPC) systems (Supplementary Note 2). FAST5 is a hierarchical data format 5 (HDF5) file with a specific schema defined by ONT. HDF5 is a generic file format for storing large data that can only be read and written using a single software library first developed in 1998. Our analysis showed that: (1) the use of increasing numbers of parallel CPU threads resulted in a relatively small reduction in the overall run time of a typical methylation calling job (Extended Data Fig. 1a); (2) this was due to inefficient data access (file reading) rather than inefficient data processing (Extended Data Fig. 1a–d); and (3) the underlying bottleneck was a limitation in the software library for reading HDF5 files, whereby parallel input/output (I/O) requests from multiple CPU threads are serialized, preventing efficient use of parallel CPU resources (Extended Data Fig. 1e and Supplementary Note 2).

Parallel computing enables scalable analysis of large datasets and is central to modern genomics. Unfortunately, our analysis shows that the FAST5 format suffers from an inherent inefficiency that ensures, even with access to advanced HPC systems, that the analysis of nanopore signal data will be prohibitively slow (Fig. 1b). For example, with the maximum resource allocation available on Australia’s National Computing Infrastructure (among the world’s largest academic supercomputers; see Supplementary Table 2—HPC-Lustre), genome-wide DNA methylation profiling on a ~30× human genome dataset runs for more than 14 days. Moreover, given that the vast majority (>90%) of the overall run time is spent simply reading FAST5 files, the performance benefits of further software optimization would be small compared to the time taken for file reading.

To overcome the inherent limitations in FAST5 format, we created SLOW5, a file format designed for efficient, scalable analysis of nanopore signal data (Fig. 1b). SLOW5 encodes all information found in FAST5 but is not dependent on the HDF5 library required to read FAST5 files. The human readable version of SLOW5 format is a tab-separated values (TSV) file encoding metadata and time series signal data for one nanopore read per line, with global metadata stored in a file header (Table 1 and Supplementary Note 3). Parallel file access is facilitated by an accompanying binary index file that specifies the position of each read (in bytes) within the main SLOW5 file (Supplementary Note 3). SLOW5 can be encoded in human readable ASCII format or a compact and efficient binary format, BLOW5, which is analogous to the seminal SAM/BAM format for storing sequence alignments^21^. The binary format optionally supports compression with zlib and ‘vbz’ (Z-standard + StreamVByte) algorithms, thereby minimizing the storage footprint while permitting efficient parallel access (Methods).

**Table 1:** Example of a SLOW5 ASCII file with a single read group

#slow5_version	1.0.0							
#num_read_groups	1							
@asic_id	0004A30B00232BEC							
@exp_start_time	2020-01-01T00:00:00Z							
@flow_cell_id	FAH00000							
@run_id	855cdb							
…	…							
#char*	uint32_t	double	double	double	double	uint64_t	int16_t*	…
#read_id	read_group	digitisation	offset	range	sampling_rate	len_raw_signal	raw_signal	…
read0	0	8192	6	1467.6	4000	123456	498,492,…	…
read1	0	8192	5	1467.6	4000	2000	491,491,…	…
…	…	…	…	…	…	…	…	…
readN	0	8192	3	1467.6	4000	3000	400,400,…	…

A SLOW5 file contains a header (rows with ‘@’ and ‘#’ prefixes) that stores metadata regarding the contents of the file and the ONT experiment(s) contained within, followed by data records (rows with no prefixes) for sequencing reads, with one read per line. SLOW5 format uses tabs (‘\t’) and newlines (‘\n’) as column and row delimiters, respectively. Complete format specifications are provided in Supplementary Note 3.

BLOW5 format is smaller than FAST5 format due to simpler space allocation and reduced metadata redundancy. Comparison of equivalent files with matched compression (FAST5-zlib versus BLOW5-zlib or FAST5-vbz versus BLOW5-vbz) revealed space savings that ranged from 18% to 69%, depending on the dataset (Supplementary Table 3). The largest savings were observed for datasets with short read lengths, and this effect was independent of compression type (Extended Data Fig. 2a,b). On a ~30× human genome dataset, BLOW5 was approximately 25% smaller (Fig. 1c), equating to a reduction of ~300 GB.

To determine the performance benefits of SLOW5, we first measured data access using a small human DNA sequencing dataset of ~500,000 reads (Supplementary Table 1) on two different HPC systems (HPC-HDD and HPC-Lustre; Supplementary Table 2). The rate of SLOW5 data access (reads per second) was faster than FAST5 across the board and increased with the use of additional CPU threads, whereas FAST5 access was largely unchanged (Fig. 1d). This trend, which reflects the capacity of SLOW5 to be efficiently accessed by multiple CPU threads in parallel, was observed for SLOW5, BLOW5 and compressed BLOW5 format, with the latter exhibiting the most efficient data access (Fig. 1d). As a result, we observed substantial improvements in data access rates when using many CPUs on both HPC systems. Using 48 CPU threads on the HPC-Lustre system, ~7 h were required to read this small dataset in FAST5 format, compared to just ~13 min in compressed BLOW5 (~32-fold improvement) (Fig. 1d).

**Extended Data Fig. 3:**
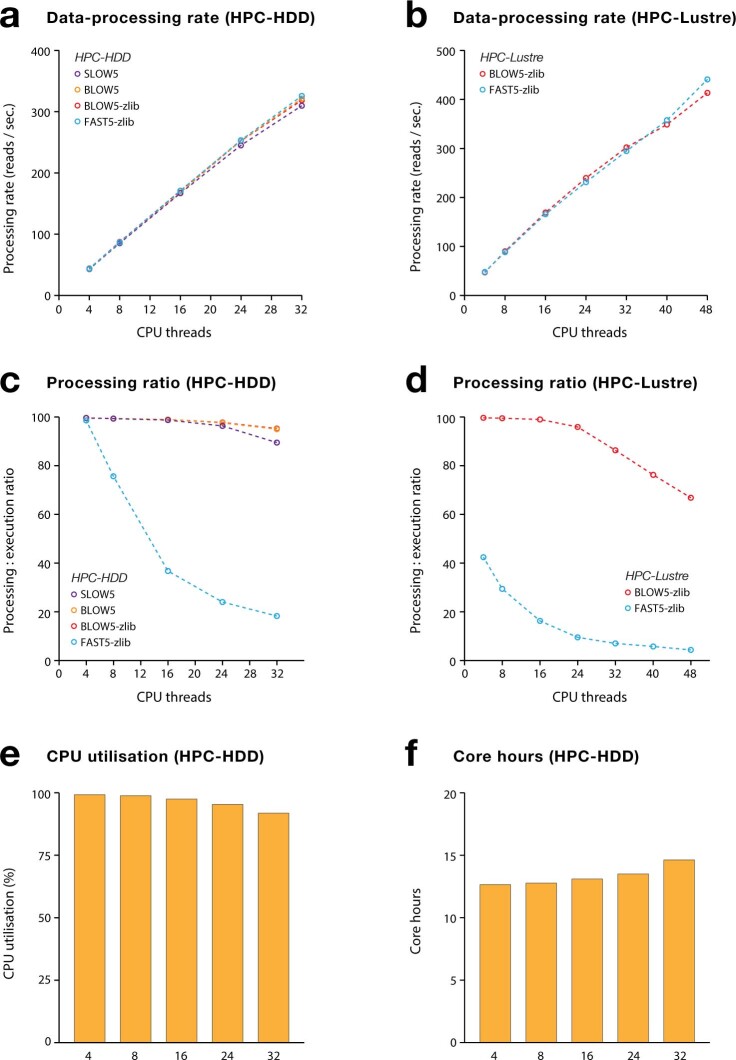
Performance metrics for DNA methylation profiling with FAST5 / SLOW5 files. (a,b) Dot plots show the rate of data processing (reads / second) during DNA methylation calling with ASCII SLOW5 (purple), binary BLOW5 (orange), BLOW5-zlib (red) and FAST5-zlib (blue) files as a function of CPU threads. Analysis was performed on two HPC architectures: HPC-HDD **(a)** or HPC-Lustre (b; see Supplementary Table 2). (c,d) Dot plots show the ratio of data-processing time relative to total execution time for the jobs above. (e,f) Bar charts show the proportional CPU utilisation **(e)** and total core hours **(f)** during the DNA methylation calling with BLOW5-zlib on the HPC-HDD system. The definition of core-hours is provided in the Methods section.

This improvement in data access manifested in performance gains during DNA methylation profiling. When using SLOW5 input, the Nanopolish/f5c runtime was reduced in proportion to the number of CPUs available (Fig. 1e). This is indicative of efficient parallel computation and was not observed when using FAST5 (Fig. 1e). As a result, substantial improvements were observed when using many CPUs, with a maximum ~15-fold reduction in runtime with 48 CPUs on the HPC-Lustre system (Fig. 1e). The improvement is the result of efficient data access, with no difference observed in data processing among the different file formats (Extended Data Fig. 3a,b). Whereas data access was the major bottleneck during FAST5 analysis, it constituted a negligible fraction of the total run time during SLOW5 analysis (Extended Data Fig. 3c,d). Put simply, this means that overall performance is dictated by the efficiency of the program rather than the time taken to read the input data, thereby enabling optimization through further engineering. For example, using GPU acceleration available in f5c^20^ with compressed BLOW5 input, we ran methylation profiling on a 30× human genome in ~10.5 h with 48 threads (>30-fold improvement compared to standard analysis with FAST5) (Supplementary Table 2).

Although the SLOW5 format is designed for scalable analysis on HPC systems, we reasoned that improved data access would be beneficial on almost any computer. To test this, we benchmarked DNA methylation profiling, as above, on a range of architectures (Supplementary Table 2). In all cases, the time consumed by data access was reduced, leading to improvements in overall execution time (Fig. 1f). As expected, improvements were greatest on systems with larger numbers of CPUs, such as a cloud-based virtual machine on Amazon AWS (~7-fold improvement at 32 CPU threads). However, benefits were observed even on miniature devices for portable computing, such as an Nvidia Xavier embedded module (~60% improvement) (Fig. 1f). In summary, SLOW5 delivered performance improvements during methylation profiling on a diverse range of hardware.

**Extended Data Fig. 4:**
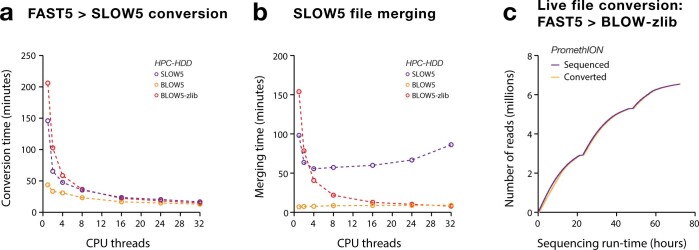
FAST5 to SLOW5 data conversion performance. **(a)** Dot plot shows the time take to convert a downsampled human genome sequencing dataset of 500,000 reads (see Supplementary Table 1) from FAST5 format to ASCII SLOW5 (purple), binary BLOW5 (orange) and compressed BLOW5-zlib (red) formats as a function of CPU threads used on the HPC-HDD system (see Supplementary Table 2). Parallel file conversion was achieved using a multi-processing approach described in the Methods section. **(b)** Dot plot shows the time taken to merge the individual files from **(a)** into a single SLOW5/BLOW5 file. **(c)** Curves show the progress of data generation (purple) and FAST5 to BLOW5-zlib conversion (orange) during a sequencing run on an ONT PromethION device with live conversion enabled. As evident, all reads are converted within minutes of availability and the entire dataset is converted to BLOW5-zlib format at the sequencing run completion.

To ensure that FAST5 to SLOW5 file conversion is not a barrier to SLOW5 adoption (given that ONT devices currently write data in FAST5 format), we implemented software (slow5tools) for efficient, parallelizable, loss-less conversion from FAST5 to SLOW5 (Methods). File conversion times are proportionally reduced with high CPU availability and are trivial compared to execution times for typical FAST5 analysis (Extended Data Fig. 4a,b). For example, conversion of a ~30× human genome dataset from FAST5 to compressed BLOW5 takes just ~3 h with 48 CPUs. We additionally implemented software for live FAST5 to SLOW5 file conversion during a sequencing run, using the internal computer on an ONT PromethION device (Extended Data Fig. 4c). This means that the user can obtain raw data in compressed BLOW5 format with effectively zero additional workflow hours required for file conversion.

The inefficiency of FAST5 data access creates delays and expenses, limiting the feasibility of ONT sequencing for many applications in research and clinical genomics. Arguably, these frictions also discourage the development of bioinformatics software that directly accesses nanopore signal data. This is in stark contrast to the simple, efficient and open-source SAM/BAM sequence alignment format, developed in 2009 (ref. ^21^), which was a key catalyst in the growth of genome informatics.

The SLOW5 format provides the framework for efficient, parallelizable analysis of nanopore signal data for any intended application. SLOW5 reading and writing is managed by efficient software application programming interfaces (APIs) for both the C (slow5lib) and Python (pyslow5) languages (Methods). This facilitates integration of SLOW5 into third-party software, including with existing packages, by replacing the existing FAST5 API. Notably, just ~70 lines of code were required for adoption of SLOW5 by the third-party software Sigmap^22^, compared to ~2,600 lines of code for FAST5 access within the same tool. This shows the simplicity of the SLOW5 API, which is fully open source and not dependent on the HDF5 library required to read FAST5. Along with the simple, intuitive structure of SLOW5 format, this will support active and open software development for nanopore data analysis.

## Methods

### Reading and writing SLOW5 files with slow5lib and pyslow5

Slow5lib (https://hasindu2008.github.io/slow5lib/) is implemented using the C programming language. To maximize portability, the slow5lib code follows the C99 standard with X/Open 7 POSIX 2008 extensions. Sequential access to SLOW5 ASCII files and SLOW5 binary files is performed using the getline() and fread() functions, respectively. For performing random disk accesses to SLOW5, the SLOW5 index is first loaded to a hash table in RAM. The read identifier serves as the hash table key. For a given read identifier, the file offset and the record length are obtained from this hash table, and pread() system call is used to load the record to the memory. Pread() allows multiple threads to perform I/O on the same file descriptor in parallel without any locking.

Pyslow5 (https://hasindu2008.github.io/slow5lib/pyslow5_api/pyslow5.html) is a Python wrapper built on top of slow5lib (interfaced using Cython) to allow easy access to SLOW5 for Python programmers.

### BLOW5 file compression

Currently, three separate compression/decompression schemes have been implemented in slow5lib, namely: (1) Z-Library (zlib, also referred to as gzip or DEFLATE*)*, which is an established library that is available by default on almost all systems; (2) Zstandard (zstd), which is a recent, open-source compression algorithm developed by Facebook; and (3) StreamVByte (svb), which is a recent integer compression technique that uses Google’s Group Varint approach^23^. Zlib and zstd are used for compressing SLOW5 records (a record is the collection of all primary and auxiliary fields of a particular read), whereas svb is for compressing the raw signal field alone. Our implementation supports first compressing the raw signal using svb and then compressing the SLOW5 record (now with the raw signal that svb compressed) using zlib or zstd, at the user’s discretion. Each read is compressed/decompressed independently from one another by using an individual compression stream for each read. Thus, multiple reads can be accessed and decompressed in parallel using multiple threads.

The use of zstd on top of svb compression is equivalent to ONT’s custom ‘vbz’ scheme (https://github.com/nanoporetech/vbz_compression), which uses these two open-source algorithms for FAST5 compression. For simplicity, we have adopted the ‘vbz’ terminology in this paper. However, we are careful to acknowledge the developers of the underlying algorithms, and slow5lib and slow5tools treat these as separate utilities. We also note that slow5lib was designed such that any other suitable compression scheme can be easily integrated if necessary, making it future proof.

### FAST5/SLOW5 conversion with slow5tools

Slow5tools (https://github.com/hasindu2008/slow5tools) is implemented on top of slow5lib using the C/C++ programming language and follows ISO C++ 2011 standard. Both slow5lib and slow5tools support Unix systems (Linux and MacOS) or even Windows using the Windows subsystem for Linux. They can be compiled using GNU C/C++ compiler (gcc/g+*+*), LLVM C/C++ compiler (clang/clang++) or Intel C/C++ Compiler (icc/icpc). We have thoroughly tested both slow5lib and slow5tools on older systems (for example, Ubuntu 14) as well as modern systems (Ubuntu 20). We have also tested both slow5lib and slow5tools on Intel, AMD and ARM (both 32-bit and 64-bit) processors.

The fast5toslow5 (f2s) and slow5tofast5 (s2f) modules in slow5tools were implemented using a heavy multi-process approach (described in Supplementary Note 2) to circumvent the HDF5 multi-threading bottleneck, whereas other modules in slow5tools, such as view, merge and split, were implemented using lightweight POSIX threads.

### SLOW5 benchmarking experiments

The benchmarking datasets described in Supplementary Table 1 were generated by sequencing genomic DNA from the human NA12878 reference sample on an ONT PromethION device. Unsheared DNA libraries were prepared using the ONT LSK109 ligation library prep, and two flow cells were used to generate ~30× genome coverage. All benchmarking experiments were performed using multi-FAST5 files, as generated by MinKNOW (distribution v.20.06.9, core v.4.0.3, and configuration v.4.0.13). FAST5 files were originally generated with zlib compression. For benchmarking experiments where FAST5-vbz files were used, these were created using ONT’s file compress_fast5 tool (v.4.0.0), which is part of the ont_fast5_api (https://github.com/nanoporetech/ont_fast5_api).

Although slow5tools is compatible with single-FAST5 format, meaning these can be easily converted to SLOW5 format, we did not consider single-FAST5 files during the benchmarking experiments described above. Data access to single-FAST5 format is slower than multi-FAST5 format because the many file-opening and file-closing operations are computationally expensive. Similarly, single-FAST5 files are larger than multi-FAST5 files due to greater metadata redundancy. We, therefore, chose not to consider single-FAST5 format here, because it would exaggerate the performance benefits of SLOW5. Given that single-FAST5 format is no longer supported by ONT, this is a reasonable omission.

To perform computational benchmarking experiments at realistic workloads, we integrated slow5lib to f5c v.0.2 CPU version, which is a restructured version of Nanopolish that enables accurate measurement of the time for each individual component of a methylation calling job. FAST5 benchmarks were performed using the same version of f5c that uses HDF5 (v.1.10.4) built with the threadsafe option enabled (see ‘Data availability’ and ‘Code availability’). POSIX threads are used in f5c to perform multi-threaded access to FAST5 and SLOW5.

To obtain FASTQ files for methylation calling, Guppy 4.0.11 was used for base-calling under the dna_r9.4.1_450bps_hac_prom base-calling profile. To obtain the BAM file for methylation calling, the reads were mapped to the hg38 reference genome (with no alternate contigs) using minimap2 v.2.17-r941 (with -x map-ont -a --secondary = no options) and sorted using SAMtools v.1.9.

Measurements and calculations were performed as follows:

1. The overall execution time (wall clock time) and the CPU time (user mode + kernel mode) of the program were measured by running the program through the GNU time utility in Linux.
2. The CPU utilization percentage is computed as:
  $${\mathrm{cpu}}\_{\mathrm{utilisation}} = {\mathrm{cpu}}\_{\mathrm{time}}/({\mathrm{execution}}\_{\mathrm{time}} \times {\mathrm{n}}\_{\mathrm{cpu}}\_{\mathrm{threads}}) \times 100$$
3. Note that this CPU utilization percentage is a normalized value based on the number of CPU threads that the program was executed with.
4. Execution time for individual components (I/O operations and data processing) was measured by inserting gettimeofday() function calls into appropriate locations in the software source code. To prevent the operating system disk cache from affecting the accuracy of I/O results, we cleared the disk cache (pagecache, dentries and inodes) each time before a program execution except on the NCI cluster where this was not permitted. On NCI, disk cache could not be cleaned as we did not have root access, so we implemented a custom program that writes and reads back hundreds of gigabytes of data (several times the size of RAM) to the storage after each experiment so the cache is filled with these mock data. Despite the effect of the hardware disk controller cache (8 GB) being negligible due to the large dataset size (>100 GB), we still executed a mock program run before each experiment.
5. ‘Core-hours’ is calculated as the product of the number of processing threads employed and the number of hours (wall clock time) spent on the job. This metric is inspired by the metric ‘man-hours’ used in the labor industry and is used in the cloud computing domain to calculate the data processing cost. In an ideally parallel program, this metric remains constant with the number of cores and threads.
6. The disk usage for different files was measured using the du command.

### Reporting Summary

Further information on research design is available in the Nature Research Reporting Summary linked to this article.

## Online content

Any methods, additional references, Nature Research reporting summaries, source data, extended data, supplementary information, acknowledgements, peer review information; details of author contributions and competing interests; and statements of data and code availability are available at 10.1038/s41587-021-01147-4.

## Supplementary information

Supplementary Information Supplementary Tables 1–3 and Notes 1–3.

Reporting Summary

## Data Availability

Datasets used in benchmarking experiments are described in Supplementary Table 1 and are available in the NCBI Sequence Read Archive (SRA) at Bioproject PRJNA744329. External datasets used in file size comparisons are publicly available at various SRA accessions, as detailed in Supplementary Table 3. Source data are provided with this paper.

## Source data

Source Data Fig. 1

## Code availability

The SLOW5 format and all associated software are free and open source. SLOW5 format specification documents can be accessed at https://hasindu2008.github.io/slow5specs. Slow5lib and pyslow5 can be accessed at https://hasindu2008.github.io/slow5lib/. Slow5tools can be accessed at https://hasindu2008.github.io/slow5tools/. Custom branches of f5c used to measure internal operation times during benchmarking experiments are available at https://github.com/hasindu2008/f5c/tree/slow5-ioprof and https://github.com/hasindu2008/f5c/tree/fastt-ioprof.
