# Supplementary material for: Fast nanopore sequencing data analysis with SLOW5
Source: Nat Biotechnol. 2022 Jan 3;40(7):1026–9. doi: 10.1038/s41587-021-01147-4 (PMC9287168; doi:10.1038/s41587-021-01147-4)
Supplement: Supplementary file 1 — Supplementary Tables 1–3 and Notes 1–3. [file 41587_2021_1147_MOESM1_ESM.pdf]

nature portfolio

https://doi.org/10.1038/s41587-021-01147-4

Supplementary information

Fast nanopore sequencing data analysis with SLOW5

In the format provided by the authors and unedited

28 October 2021

SUPPLEMENTARY INFORMATION

Hasindu Gamaarachchi, Hiruna Samarakoon, Sasha P. Jenner, James M. Ferguson, Timothy G. Amos, Jillian M. Hammond, Hassaan Saadat, Martin A. Smith, Sri Parameswaran, Ira W. Deveson

Kinghorn Centre for Clinical Genomics, Garvan Institute of Medical Research, Sydney, NSW, Australia.

LIST OF SUPPLEMENTARY MATERIALS

Supplementary Table 1. Datasets used for benchmarking experiments. **p.1**

Supplementary Table 2. Specifications of all computers used in this study. **p.2**

Supplementary Table 3. FAST5 vs BLOW5 file size comparison across various datasets. **p.3**

Supplementary Note 1. FAST5 format de-mystified. **p.4**

Supplementary Note 2. A limitation in FAST5 files prevents efficient parallel analysis. **p.18**

Supplementary Note 3. SLOW5 format specifications (version 0.2.0). **p.23**

Supplementary Table 1. Datasets used for benchmarking experiments.

Dataset	Platform	Pore	Reads	Total seq. (Gbases)	Size FAST5-zlib	Size FAST5-vbz	Size SLOW5	Size BLOW5	Size BLOW5-zlib	Size BLOW5-vbz
~30X human genome (NA12878)	PromethION	R9.4.1	9,083,052	93.4	1.3 TB	978 GB	4.0 TB	2.0 TB	1.0TB	707 GB
Downsampled human dataset (NA12878)	PromethION	R9.4.1	500,000	5.1	71 GB	51 GB	212 GB	106 GB	53 GB	36 GB

Supplementary Table 2. Specifications of all computers used in this study.

System	Type	CPU	CPU cores	RAM (GB)	GPU	File system	Disk System	OS
HPC-HDD	HPC with HDD RAID	2 × Intel Xeon Gold 6154	36	384	-	ext4	12×10TB HDD drives with RAID6 configuration	Ubuntu 18.04.3 LTS
HPC-Lustre	NCI CPU node with distributed lustre file system	2 x core Intel Xeon Platinum 8274	48	192	-	lustre	7200 4TB disks in 120 NetApp disk arrays	CentOS 8.3.2011
HPC-GPU	NCI GPU node with distributed lustre file system	2 x Intel Xeon Platinum 8268	48	384	Nvidia Tesla V100-SXM2-32GB (Volta architecture)	lustre	7200 4TB disks in 120 NetApp disk arrays	CentOS 8.3.2011
Cloud-FsX	Amazon AWS c5a.16xlarge instance with distributed lustre file system	AMD EPYC 7R32 (virtual machine)	32 virtual machine	128	-	Amazon FSx for Lustre	Amazon FSx Lustre storage (persistent HDD)	Ubuntu 20.04.2 LTS
Cloud-EBS	Amazon AWS c5a.16xlarge instance with elastic block storage (EBS)	AMD EPYC 7R32 (virtual machine)	32 virtual machine	128	-	ext4	1x500GB elastic block storage (standard magnetic)	Ubuntu 20.04.2 LTS
Workstation-SSD	GPU Workstation with SSD	1xAMD Ryzen Threadripper 3970X	32	128	NVIDIA 3090 - 24GB (Ampere architecture)	ext4	4x500GB SSD drives with RAID0 configuration	Ubuntu 18.04.5 LTS
Workstation-NFS	Workstation with network file system (NFS)	1xAMD Ryzen Threadripper 3970X	32	128	-	ext4 mounted as NFS	12x12 TB HDD with RAID 10 configuration (Synology DS3617xs NAS)	Ubuntu 18.04.5 LTS
Laptop-HDD	Dell XPS 9570 laptop with a USB 3.0 external HDD attached	Intel core i7-8750H CPU	6	16	NVIDIA 1050 Ti - 4 GB (Pascal architecture)	ext4	1x 500GB HDD (external USB3.0 drive)	Ubuntu 18.04.1 LTS
Embedded system	Jetson Xavier AGX with a USB 3.0 external HDD attached	ARM v8 64-bit CPU	8	16	NVIDIA tegra - 16GB shared with RAM (Volta architecture)	ext4	1x 500GB HDD (external USB3.0 drive)	Ubuntu 18.04.3 LTS

Supplementary Table 3. FAST5 vs BLOW5 file size comparison for various datasets.

Dataset	Source	Type	FAST5-zlib (GB)	BLOW5-zlib (GB)	Saving (%)
**NA12878_prom**	Internal (NCBI: SRR15058166)	~30X human genome (NA12878)	1330	1012	23.95
**NA12878_prom_sub**	Internal (NCBI: SRR15058164)	Downsampled human dataset (NA12878)	70	53	24.57
**PUXP097306**	Internal	Ultra-long human gDNA sequencing	346	281	18.58
**PBXP153375**	Internal	Sheared human gDNA sequencing	1646	1212	26.34
**PQXT038257**	Internal	Q20+ human gDNA sequencing	522	417	20.12
**MBXM172390**	Internal	ReadFish targeted human gDNA sequencing	268	205	23.25
**PBXP088299**	Internal	Human cDNA sequencing	3881	1495	61.49
**PNXP021236**	Internal	Mouse direct-RNA sequencing	481	395	17.83
**PLPL079987**	Internal	Viral PCR amplicon sequencing	338	105	69.04
**PUBLIC-GENOME**	NCBI: SRR14493367	Standard human gDNA sequencing	1244	931	25.15
**PUBLIC-METAGENOME**	NCBI: ERR4991716	Metagenome amplicon sequencing	18	8	54.53
**PUBLIC-TRANSCRIPTOME**	NCBI: SRR13261194	Human direct-RNA sequencing	9	7	22.96
**PUBLIC-PLANT**	NCBI: SRR15611015	Datura stramonium plant genome sequencing	27	19	29.55
**PUBLIC-VIRAL**	ARTIC community	SARS-CoV-2 amplicon sequencing	21	8	61.34
				**average**	**34%**
				**median**	**25%**
				**range**	**18-69%**

Supplementary Note 1. FAST5 format de-mystified

PREAMBLE

FAST5 files are Hierarchical Data Format 5 (HDF5) files with a specific schema defined by Oxford Nanopore Technologies (ONT) for storing raw current-signal data generated from ONT devices. We have compiled the following material to help researchers to understand FAST5 files, and consulted ONT on various definitions within their format. This document should not be interpreted as a definitive specification document from the developers of FAST5, although it is the most detailed description of FAST5 format that we are aware of.

There are two FAST5 types: single-FAST5 and multi-FAST5 (first appearing around September 2018). A multi-FAST5 file contains a batch of reads in a single file whereas a single-FAST5 file contains just a single read per file. Single-FAST5 format is no longer used by ONT. In this document, FAST5 will always refer to multi-FAST5 unless otherwise stated.

To read FAST5 files we use the HDF5 library and HDF5 tools [1].

BASICS

A FAST5 (HDF5) file is like a file system. Just as there are multiple levels of ***directories*** and ***files*** in a file system, a FAST5 (HDF5) file contains ***groups*** and ***datasets***, respectively. The term **HDF5 objects** is an umbrella term for both groups and datasets. HDF5 objects can optionally contain ***attributes***, which are key-value pairs. HDF5 related terms are defined below with examples.

Groups

HDF5 groups (and links[1]) organise HDF5 objects. Every HDF5 file contains a root group that can contain other groups or links to other HDF5 objects[2]. Working with groups and group members (HDF5 objects) is similar in many ways to working with directories and files in UNIX. As with UNIX directories and files, objects in an HDF5 file are often described by giving their full (or absolute) path names.

- */* signifies the root group.
- */foo* signifies a member of the root group called *foo*.
- */foo/zoo* signifies a member of the group *foo*, which in turn is a member of the root group.

Datasets

HDF5 datasets organise and contain the actual data values [2]. A dataset consists of metadata (datatype, datasize, compression technique, etc) that describes the data, in addition to the data itself. In any read within a FAST5 file, two datasets are found; the *Raw* group contains the raw current-signal the *Analyses* group contains the FASTQ data (only if live base-calling was enabled) [1].

[1] Links are like directory/file paths in a file system. Links can be absolute, relative or even symbolic.
[2] Other objects can, in theory, be in the same FAST5 file or in a different FAST5 file.

Attributes

Attributes can optionally be associated with HDF5 objects. The attributes have two parts: a name and a value. Attributes are accessed by opening the object that they are attached to; hence, they are not independent objects. Typically an attribute is small in size and contains details about the object that it is attached to.

Attributes look similar to HDF5 datasets in that they have metadata such as data type and dataspace. However, unlike HDF5 datasets, HDF5 attributes do not support partial I/O operations and cannot be compressed or extended [2].

HDF5 dataspace

The HDF5 *dataspace* must be defined prior to defining an HDF5 dataset or an attribute. The dataspace defines some metadata such as the size and shape of the dataset or attribute raw data (i.e., the number of dimensions and the size of each dimension of the multidimensional array in which the raw data is represented) [2].

HIERARCHY OF A MULTI-FAST5 FILE

The root group (e.g. /fmh_15… in the snapshot below) contains a group for each read that is named as "read_" followed by the read identifier (e.g. read_001f4… in the snapshot below):

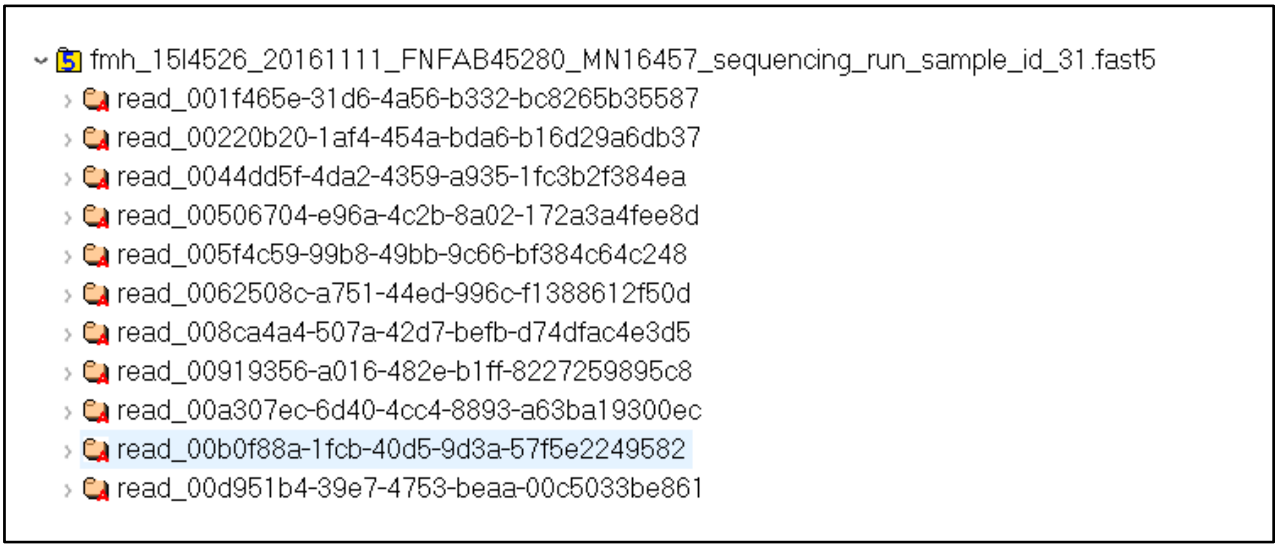

Under each read group, there are the following groups:

- Raw
- channel_id
- context_tags
- tracking_id
- Analyses

Note that the *Analyses* group is only available in base-called FAST5 files. The groups found in a FAST5 file that has not been base-called are shown in the snapshot below:

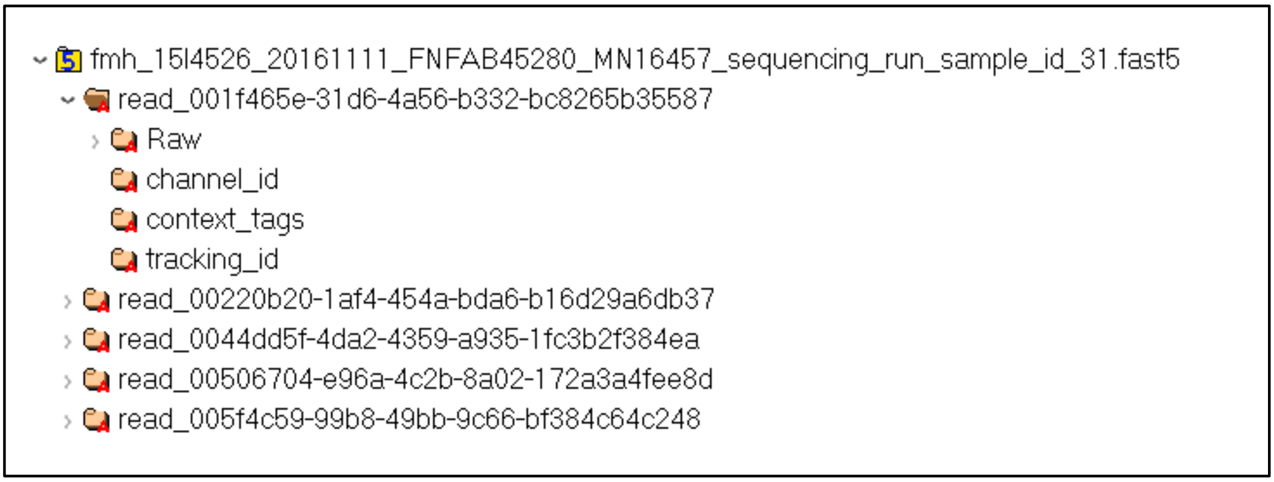

As the name suggests, *Raw* contains the raw signal (raw data acquisition values) and associated metadata. *channel_id* contains (but is not limited to) parameters useful for converting the raw signal into pico-ampere values. *context_tags* and *tracking_id* contain global information that are common to the sequencing run. More information on these groups is provided below.

The *Analyses* group is for storing data resultant from various downstream analyses, such as base-calling. For instance, if the Guppy base-caller is run with the option to output base-called FAST5 files, those output FAST5 files will contain this *Analyses* group. *Analyses* groups can be used by custom software (e.g. Tombo) for storing data from additional downstream analyses.

In the following subsections we provide detailed descriptions of groups mentioned above, except the *Analyses* group. Since we are concerned with the raw signal data, basecalled data is not in the scope of this document.

Root_group

Root group has two attributes *file_type* (note that *file_type* is only available in multi-fast5 from version 2.2 onwards) and *file_version*. Note that we do not make any assumptions about file-structure based on FAST5 version numbers, because we have observed some inconsistencies across different files of the same version, and we would discourage users from doing so.

1. **file_type**
   Example value: multi-read
   Data type: String
2. **file_version**
   Example value: 2.2
   Data type: String

Read

A read group has two attributes *run_id* and *pore_type* (note that *pore_type* is not available in multi-fast5 v2.0).

1. **run_id**
   The value of this attribute is constant across all the read groups.
   Example value: fe697f519ab04ba540bc4fe93f7cbd86669f38ca
   Data type: String
2. **pore_type**

In existing FAST5 versions, this value is empty. This attribute may be used in the future to distinguish different pore types within a single flow cell.
Example value: <not set>
Data type: String

Raw

Raw group contains one dataset and seven attributes. The dataset is the raw signal, which is a series of 16-bit integers (HDF5 Datatype = *H5T_STD_I16LE*). These are the integer values directly coming from the data acquisition process (analog to digital converter). This raw signal can be converted into pico Ampere (pA) values using attributes available in the *channel_id* group (explained later).

The seven attributes from the *Raw* group are listed below with a description of each attribute, example values and the data type. Understanding these descriptions require a brief understanding of an ONT flow cell. A flow cell has multiple channels allowing multiple DNA/RNA strands to be sequenced in parallel. For instance, a MinION flow cell has 512 channels and thus can sequence 512 strands in parallel. Each channel contains one or more wells[3]. For instance, a MinION flow cell has 4 wells per channel. The wells within a channel are connected to a multiplexer (MUX), a switch that controls which of the four wells in the channel is controlled and read out by the circuits. Please refer to reference [3] or [4] for more information about channels and multiplexers.

Note that some of the information below is extracted from ONT document [1].

1. **read_number**
   A unique number within each channel counted upwards from zero [1]. Note that not all reads generated are "strand" reads, but only strand reads are written to the final fast5 file, so some read numbers may be absent.
   Example value: 17981
   Data type: 32-bit signed integer
2. **read_id**
   A unique identifier for the read. This is a Universally unique identifier (UUID) version 4 and should be unique for any read from any device.
   Example value: 00592138-f120-4ab5-9916-c5567adb8e29
   Data type: String
3. **start_time**
   The start time of the read. The unit for *start_time* is 'number of signal samples', so *start_time* has to be divided by sampling rate (Read_xxxx/channel_id/sampling_rate) to get the start time in seconds (i.e. the time since the run was started).
   Example value: 335845487
   Data type: 64-bit unsigned integer
4. **duration**
   The duration of the read. The unit for *duration* is also 'number of signal samples'.
   Example value: 1467
   Data type: 32-bit unsigned integer
5. **start_mux**

[3] Each well should ideally contain one pore

The MUX setting[4] for the channel when the read began. Due to timing issues this can sometimes reflect what the MUX was just *before* the read began; this will only matter for reads that start immediately after a MUX change.

Example value: 4

Data type: 8-bit unsigned integer

6. **median_before**

The estimated median current level immediately preceding the read. In most cases this can be used as an estimate of the open pore level[5].

Example value: 238.78225708007812

Data type: 64-bit floating-point

7. **end_reason**

This is a new attribute in FAST5 v2.2 onwards.

Example value: unblock_mux_change

Data type: 8-bit enum

```
ATTRIBUTE "end_reason" {
    DATATYPE  H5T_ENUM {
    H5T_STD_U8LE;
    "unknown"       0;
    "partial"       1;
    "mux_change"            2;
    "unblock_mux_change" 3;
    "signal_positive"  4;
    "signal_negative"  5;
    }
```

Channel_id

This group has attributes that are relevant to the channel that sequenced a given read. The *channel_id* group has the following attributes.

1. **channel_number**

The channel number from which the read was acquired.

Example value: 504

Data type: String

2. **digitisation**

The digitisation is the number of quantisation levels in the Analog to Digital Converter (ADC). That is, if the ADC is 12 bit, digitisation is 4096 (2^12).

Example value: 8192.0

Data type: 64-bit floating-point

3. **offset**

The ADC offset error. This value is added when converting the signal to pico ampere.

Example value: 10.0

Data type: 64-bit floating-point

4. **range**

The full scale measurement range in pico amperes.

Example value: 1441.389892578125

[4] out of the wells in the channel, which well the mux is set to sequence

[5] open-pore state is when there is no strand inside the pore

Data type: 64-bit floating-point

5. **sampling_rate**

   Sampling frequency of the ADC, i.e., the number of data points collected per second (in Hertz).

   Example value: 4000

   Data type: 64-bit floating-point

Of these attributes, *digitisation*, *offset* and *range* can be used to transform the raw signal in the *Raw* group (raw ADC values), to pico-ampere current values as follows:

```
signal_in_pico_ampere = (raw_signal_value + offset) * range / digitisation
```

Context_tags

The *context_tags* group has global attributes that describe the sequencing run. The attributes under the *context_tags* group are listed below with short descriptions. The data type of the value of all the attributes listed under *context_tags* group is String.

1. **barcoding_enabled**

   Indicates if barcode demultiplexing is enabled during live basecalling

   Example value: 0

2. **experiment_duration_set**

   Indicates the duration of the experiment selected when starting the sequencing run (in minutes)

   Example value: 4320

3. **experiment_type**

   Indicates the type of the experiment, for instance, *genomic_dna* or *rna*.

   Example value: genomic_dna

4. **local_basecalling**

   Indicates if live base calling is enabled or not (set to 1 or 0).

   Example value: 1

5. **package**

   This attribute relates to the Bream package [https://github.com/nanoporetech/minknow_lims_interface]

   Example value: bream4

6. **Package_version**

   Example value: 6.0.7

7. **sample_frequency**

   Typically the same as the *sampling_frequency* in the *channel_id* group

   Example value: 4000

8. **sequencing_kit**

   The sequencing kit selected by the user in the GUI, for instance, *sqk-lsk109* or *sqk-rna002*. [https://store.nanoporetech.com/sample-prep.html]

   Example value: sqk-lsk109

There can be additional attributes such as *basecall_config_filename*, depending whether live basecalling was turned on/off when the sequencing run was started.

Tracking_id

The *tracking_id* group has global attributes relevant to the sequencing run and the sequencing device. These are mostly for internal use by ONT, who have assisted us in providing the definitions below. The data type of the value of all the attributes listed under *tracking_id* group is String.

1. **asic_id**

   Application Specific Integrated Circuit identifier (ASIC) of the flow cell (unique number of the chip). Enables tracking of batches of chips.

   Example value: 213553007

2. **asic_id_eeprom**

   Identifier of the ASIC's electrically erasable programmable read-only memory (EEPROM) of the flow cell.

   Example value: 5309577

3. **asic_temp**

   The temperature in degrees celsius of the ASIC chip at the start of the sequencing run.

   Example value: 28.867193

4. **asic_version**

   The version of ASIC being used.

   Example value: IA02D

5. **auto_update**

   Whether auto update in Minknow is enabled or not.

   Example value: 0

6. **auto_update_source**

   The link to the Minknow update source.

   Example value: https://mirror.oxfordnanoportal.com/software/MinKNOW/

7. **bream_is_standard**

   Bream is one of the software for controlling sequencing.

   Example value: 0

8. **configuration_version**

   The version of the configuration system in MinKNOW including the experiment scripts.

   Example value: 4.0.13

9. **device_id**

   The serial ID of the MinION or device position for GridION/PromethION.

   Device position on GridION/PromethION refers to the ID of the bay (slot where the flowcell is put) on the device.

   Example value: X2

10. **device_type**

    The device type, that is whether MinION, PromethION or GridION.

    Example value: gridion

11. **distribution_status**

    Stable vs dev/alpha/beta status.

    Example value: stable

12. **distribution_version**

    MinKNOW version.

    Example value: 20.06.9

13. **exp_script_name**

The name of the experiment script run along with optional parameters passed to it, based on what kits are selected in MinKNOW for sequencing.
Example value: sequencing/sequencing_MIN106_DNA:FLO-MIN106:SQK-LSK109

14. **exp_script_purpose**
The 'purpose' of the experiment script. For example, whether the experiment was a real sequencing run or a simulation playback.
Example value: sequencing_run
15. **exp_start_time**
Start time of sequencing run in ISO 8601 standard.
Example value: 2020-09-08T01:23:21Z
16. **flow_cell_id**
Unique ID for the flowcell, used by ONT to track flowcell metrics and warranty.
Example value: FAN43349
17. **flow_cell_product_code**
The type of flowcell (product code of the flowcell and pore type). These will be different based on R9.4.1, R10.3, R9.5, PromethION, etc.
Example value: FLO-MIN106
18. **guppy_version**
Guppy version being used by MinKNOW.
Example value: 4.0.11+f1071ce
19. **heatsink_temp**
The temperature (in degrees celsius) of the heat sink on the ASIC at the start of the sequencing run.
Example value: 33.996094
20. **hostname**
The hostname of the computer/machine doing the sequencing run.
Example value: GXB02243
21. **installation_type**
This is the MinKNOW installation type.
Example value: nc
22. **local_firmware_file**
Example value: 1
23. **operating_system**
The operating system and version of the computer performing the sequencing run.
Example value: ubuntu 16.04
24. **protocol_group_id**
This is the unique ID given to the group of acquisition periods during a run, denoted by run_id. Multiple acquisition periods can occur during a single "run", depending on the protocol.
Example value: GLFN180082
25. **protocol_run_id**
This is a unique identifier for the experiment GROUP (just in case the name given by the user is not unique). This is the same for each run of the same experimental group.
Example value: f2c69573-5fef-43b8-8d81-9cb20634aa7c
26. **protocol_start_time**
The start time of the data acquisition periods for a *protocol_group_id*. Appeared in FAST5 2.3.

Example value: 2021-08-26T15:34:52.186021+10:00

27. protocols_version

Allows MinKNOW to track various protocols for barcoding, kits, etc.

Example value: 6.0.7

28. run_id

The unique run ID which will be different for each run (data acquisition period), even in the same experiment group. Whenever MINKNOW starts an experiment script for data acquisition, a new run_id is generated.

Example value: 07770780274b0e3703f00d969291b1a37a5a6be1

29. sample_id

Sample ID is the name given by the user for the sample.

Example value: NA12878

30. usb_config

Information about the connection between the flowcell and the computer.

Example value: GridX5_fx3_1.1.3_ONT#MinION_fpga_1.1.1#bulk#Auto

31. version

MinKNOW version.

Example value: 4.0.3

Note that the above list is not an exhaustive list. For instance, FAST5 files generated on the PromethION have additional attributes such as *hublett_board_id* and *satellite_firmware_version*.

FAST5 VERSIONS & THEIR ATTRIBUTES

The following table shows the availability (and unavailability) of attributes in un-basecalled multi-FAST5 files for different file versions.

Grey cells = attribute available.

Group	Attribute name	V2.0	V2.2	v2.3
/	file_type			
	file_version			
/read	run_id			
	pore_type			
/read/Raw	start_time			
	duration			
	read_number			
	start_mux			
	read_id			
	median_before			
	end_reason			
/read/channel_id	digitisation			
	offset			

	range			
	sampling_rate			
	channel_number			
/read/context_tags	barcoding_enabled			
	experiment_duration_set			
	experiment_type			
	local_basecalling			
	package			
	package_version			
	sample_frequency			
	sequencing_kit			
	experiment_kit			
	filename			
	user_filename_input			
/read/tracking_id	asic_id			
	asic_id_eeprom			
	asic_temp			
	asic_version			
	auto_update			
	auto_update_source			
	bream_core_version			
	bream_is_standard			
	bream_ont_version			
	bream_prod_version			
	bream_rnd_version			
	configuration_version			
	device_id			
	device_type			
	distribution_status			
	distribution_version			
	exp_script_name			
	exp_script_purpose			
	exp_start_time			
	flow_cell_id			
	flow_cell_product_code			

	guppy_version			
	heatsink_temp			
	host_product_code			
	host_product_serial_number			
	hostname			
	installation_type			
	local_firmware_file			
	operating_system			
	protocol_group_id			
	protocol_run_id			
	protocol_start_time			
	protocols_version			
	run_id			
	sample_id			
	usb_config			
	version			

CONSTANT & VARIABLE ATTRIBUTES

Many FAST5 attributes are identical amongst all the reads within a single sequencing run (within multi-FAST5 files as well as amongst different multi-FAST5 files). For example, all reads from a given experiment will have the same *run_id*. Some attributes are variable between different reads, even within a single multi-FAST5. For example, each read has a different *read_id*. All the attributes in *contex_tags* and *tracking_id* are constant across all reads in a single sequencing run, whereas most of the attributes in *Raw* and *channel_id* are variable between reads (with a few exceptions).

The variable attributes amongst all groups (except the *Analyses* group) are:

- duration
- end_reason (not in version 0.6 but in 2.2)
- median_before
- read_id
- read_number
- start_mux
- start_time
- channel_number
- offset

Note that the dataset “Signal” obviously has variable data. All the other attributes are contant.

ADVANCED INFORMATION

Symbolic links

For a given FAST5 file, the values of the attributes belonging to the two groups, *context_tags* and *tracking_id* are the same for all the reads in that FAST5 file. Hence only the first read_xxxx group has the actual attributes. The rest of the read_xxxx groups maintain symbolic links [2] to the first read_xxxx group. One can observe the linking structure of a FAST5 file using a utility program called *h5dump* developed by the HDF5 group.

The following is an example output for a FAST5 file where read_000200a4* is the first read group. As listed below the rest of the read groups' *context_tags* and *tracking _id* attributes maintain links (symbolic links) to the *context_tags* and *tracking_id* attributes of the first read group respectively. This observation was valid for all the FAST5 files we have examined.

```
GROUP "context_tags" {
        HARDLINK        "/read_000200a4-0347-4a49-b800-
37ad7b4287c9/context_tags"
        }
 GROUP "tracking_id" {
        HARDLINK        "/read_000200a4-0347-4a49-b800-
37ad7b4287c9/tracking_id"
        }
```

ONT h5 validator

[Ont_h5_validator] is a tool developed by ONT to check if a given FAST5 file complies with the FAST5 schema. This tool only considers a subset of the complete FAST5 schema to validate a file.

Single-FAST5 format fields

The groups, attributes and some example values for a single-FAST5 file are provided below for the sake of completeness, despite no longer being in use.

**PreviousReadInfo**<br>previous_read_id = cf435984-627d-450d-a81d-2a55c6060c80<br>previous_read_number = 80
**Read**<br>duration = 30695<br>median_before = 206.2032470703125<br>read_id = b3d473e9-34f0-4ad6-a030-61ba6ab458bc<br>read_number = 99<br>start_mux = 4<br>start_time = 318648
**channel_id**<br>channel_number = 707<br>digitisation = 2048.0<br>offset = -196.0<br>range = 748.5801660113588<br>sampling_rate = 4000.0
**context_tags**

experiment_duration_set = 3840
experiment_type = genomic_dna
fast5_output_fastq_in_hdf = 1
fast5_raw = 1
fast5_reads_per_folder = 4000
fastq_enabled = 1
fastq_reads_per_file = 4000
filename = pct0028_20181029_0004a30b00232bec_1_e11_h11_sequencing_run_lxbab132606_84140
flowcell_type = flo-pro002
kit_classification = none
local_basecalling = 1
local_bc_comp_model =
local_bc_temp_model = template_r9.4_450bps_5mer_raw.jsn
sample_frequency = 4000
sequencing_kit = sqk-lsk109
user_filename_input = lxbab132606

tracking_id
asic_id = 0004A30B00232BEC
asic_id_eeprom = 0004A30B00232BEC
asic_temp = 36.990513
asic_version = Unknown
auto_update = 0
auto_update_source = https://mirror.oxfordnanoportal.com/software/MinKNOW/
bream_is_standard = 0
device_id = 1-E11-H11
device_type = promethion
exp_script_name = 59dfa94107ee2b6c0f4be0822482e7da35b4116a-da65898430ab8c4bfe54ba7064f0301390b76211
exp_script_purpose = sequencing_run
exp_start_time = 2018-10-29T01:40:23Z
flow_cell_id = PAD11989
heatsink_temp = 41.996017
hostname = PCT0028
hublett_board_id = 013220e36be4c748
hublett_firmware_version = 2.0.5
installation_type = nc
ip_address =
local_firmware_file = 1
mac_address =
operating_system = ubuntu 16.04
protocol_run_id = e3b445eb-5626-48ef-acc2-b28bcc611009
protocols_version = 0.0.0.0
run_id = 855cdb4b269484b72699b681e539e090c4a50bbb
sample_id = LXBAB132606
satellite_board_id = 0000000000000000
satellite_firmware_version = 2.0.4
usb_config = firm_1.2.3_ware#rbt_4.5.6_rbt#ctrl#USB3
version = 1.14.2

REFERENCES

[1] Oxford Nanopore Technologies. Read .fast5 files from the instrument. *technical_documents* https://community.nanoporetech.com/technical_documents/data-analysis/v/datd_5000_v1_revn_22aug2016/read-fast5-files-from-th (2016).

[2] The HDF5 Group. HDF5 User's Guide: HDF5 Release 1.6.10. https://support.hdfgroup.org/HDF5/doc1.6/UG/ (2009).

[3] Lu, H., Giordano, F. & Ning, Z. Oxford Nanopore MinION Sequencing and Genome Assembly. *Genomics, Proteomics & Bioinformatics* vol. 14 265–279 (2016).

[4] Lannoy, C. de, de Lannoy, C., de Ridder, D. & Risse, J. The long reads ahead: de novo genome assembly using the MinION. *F1000Research* vol. 6 1083 (2017).

Supplementary Note 2. An inherent limitation in FAST5 files prevents efficient parallel analysis.

PREAMBLE

High Performance Computing (HPC) systems offer significant computational power through many-core CPUs that can be utilised in parallel. Moreover, HPC systems have Redundant Arrays of Independent Disks (RAID) storage composed of many disks for higher I/O throughput. Multi-threaded analysis on HPC systems is now standard practice in genomics, enabling efficient analysis of large DNA sequencing datasets.

In our experience, the analysis of nanopore signal data (FAST5 files) is generally slow, even on powerful HPC systems. To understand why, we undertook a detailed investigation of a typical signal-level ONT analysis on a typical HPC system. We selected DNA methylation (5mC) profiling with the popular software *Nanopolish* [1] as our example use-case to assess computational performance and identify potential bottlenecks.

APPROACH

We executed the *call-methylation* tool within the *Nanopolish* toolkit on a downsampled ONT human genome sequencing dataset of 500 million reads (see **Supplementary Table 1**). We used a restructured version of the *Nanopolish* software (*f5c*) that allowed us to record the wall-clock time spent on I/O operations and data processing. This enables the time consumed by individual components of the analysis (FAST5 files access, FASTA file access, BAM file access & data processing) to be monitored separately. The experiment was run on an HPC system 12 × 10TB HDD drives with RAID6 configuration (**Supplementary Table 2**), using either 4, 8, 16, 24 or 32 CPU threads.

AN I/O BOTTLENECK LIMITS PERFORMANCE

We observed a relatively modest improvement in the overall execution time with increasing numbers of CPU threads and almost no improvement beyond 16 threads (**Extended Data Fig.1a**). While we observed a linear increase in the rate of data processing with additional CPU threads, there was no increase in the rate of FAST5 data access and, hence, FAST5 data access came to represent an increasingly large fraction of the total execution time (**Extended Data Fig.1a,b**). We observed a steep decline in CPU utilisation with increasing numbers of threads, dropping to just 18% utilisation at 32 threads (**Extended Data Fig.1c**). Likewise, we found that core-hours (which should be constant in an ideal scenario; see definition under **Methods**) increased with additional threads (**Extended Data Fig.1d**).

These results clearly demonstrate that additional CPU threads are not efficiently utilised by *Nanopolish/f5c* to improve the overall execution time. There can be two possible explanations for the inability of a tool to efficiently utilise parallel resources: (i) a bottleneck in data processing; and (ii) a bottleneck in Input/Output (I/O). Our observations strongly suggest that an I/O bottleneck is the primary reason for the under-utilisation during methylation calling.

UNDERSTANDING THE BOTTLENECK

We deployed performance monitoring and profiling tools during the above experiment to elucidate the causes of inefficient resource-utilisation and performance.

Hypothesis-1: The performance of the software tool is bounded by file I/O.

We observed through the *htop* utility in Linux that the majority of *Nanopolish/f5c* threads were in the 'D' state during the experiment. The 'D' state is defined as the 'state of the process for disk sleep (uninterruptible)'. This suggests that the software is bounded by file I/O.

Hypothesis-2: The file I/O bottleneck is caused by the HDF5 library and not by the limitation of physical disks.

We observed disk usage statistics using the *iostat* utility and found that the disk system was not fully utilised during the experiment (i.e., the observed number of disk reads per second was around 100 Input/output operations per second (IOPS)), while the particular disk system could handle more than 1000 IOPS). This implies that the I/O bottleneck is not due to the limitation of physical disks to serve data fast enough to saturate the processor.

To investigate further, we profiled *Nanopolish/f5c* with *Intel Vtune* under concurrency profiling. It reveals that the majority of the 'wait time' is due to a conditional variable (synchronisation primitive) in the underlying HDF5 library that is used to access FAST5 files. Closer inspection of the HDF5 library revealed that the thread-safe version of the HDF5 library serialises the calls for disk read requests. Thus, we reasoned that CPU under-utilisation is caused by the disk requests being serialised by the HDF5 library, consequently causing a bottleneck that limits the utility of a multi-disk RAID system.

A HDF5 LIBRARY LIMITATION PREVENTS PARALLEL ACCESS

The HDF5 library required to read and write FAST5 files uses synchronous I/O calls and even the latest HDF5 implementation (HDF5-1.10) does not support asynchronous I/O[6]. This, by itself, is not an issue as multiple synchronous I/O operations can be performed in parallel using multiple I/O threads to exploit the high throughput of RAID systems (**Extended Data Fig.1e**; upper).

However, the HDF group (that maintains the HDF5 library) mentions that the thread-safe version of the HDF5 library is not thread efficient and that it effectively serialises the calls for disk read requests [2]. The global lock in the thread safe version of the HDF5 library creates this limitation, as explained in the following extract from the HDF5 documentation:

"Users are often surprised to learn that (1) concurrent access to different datasets in a single HDF5 file and (2) concurrent access to different HDF5 files both require a thread-safe version of the HDF5 library. Although each thread in these examples is accessing different data, the HDF5 library modifies global data structures that are independent of a particular HDF5 dataset or HDF5 file. HDF5 relies on a semaphore around the library API calls in the thread-safe version of the library to protect the data structure from corruption by simultaneous manipulation from different threads. Examples of HDF5 library global data structures that must be protected are the freespace manager and open file lists."

Thus, in spite of having multiple I/O threads, I/O requests for HDF5 files have to go through the HDF5 library (**Extended Data Fig.1e**; lower). In a scenario where multiple I/O threads are requesting I/O from the HDF5 library in parallel, the lock inside the HDF5 libraries serialises the parallel requests, effectively issuing only one request at a time to the operating system disk request queue.

A MORE DETAILED EXPLANATION

Extended Data Fig.1e (upper) illustrates how multiple I/O threads can be used to perform parallel disk accesses using synchronous I/O. Suppose the disk system has K disks, up to K requests may be served simultaneously depending on the RAID level; i.e., K simultaneous parallel reads are possible on a RAID 0

[6] In synchronous I/O calls, the OS, upon receiving the call, puts the user-space thread to sleep and the thread can no longer submit I/O requests until the disk reading is completed and woken by the OS. Conversely, asynchronous I/O system calls return immediately without the thread being put to sleep and the thread can continue to submit another asynchronous request.

system with *K* disks. Let *t* be the average disk request service time (from the time of the system call to when the thread is woken up).

For a program that launches *K* I/O threads and if the disk controller can serve *K* requests in parallel, the total time for *n* disk reads is $T' = t \times \frac{n}{K}$. However, in spite of having multiple I/O threads, I/O requests for HDF5 files have to go through the HDF5 library. **Extended Data Fig.1e** (lower) illustrates this, where *K* I/O threads are requesting I/O from the HDF5 library in parallel. However, the lock inside the HDF5 serialises the parallel requests, effectively issuing only one request at a time to the operating system disk request queue. The operating system will put the thread to sleep and this is equivalent to a single I/O thread. Thus, the total time spent on disk accesses *T* will be $T = t \times n$, and essentially, the high throughput capability of multiple disks in a RAID configuration is under-utilised.

POSSIBLE WORKAROUNDS

We explored several possible approaches to circumvent the FAST5 bottleneck and these are articulated below. However, due to limitations in each of these approaches we decided instead to create an alternative file format that is not dependent on the HDF5 library.

Fixing HDF5 Library

One possible solution to the limitation articulated above is to re-engineer the HDF5 library to be thread efficient. However, the HDF5 library is a complicated library with a large code base of >300,000 lines of C code and such a fix would need to be carried out by the HDF Group. The HDF Group mentions that the future plan to implement efficient multi-threaded access is currently hindered by inadequate resources [2]. Therefore, such a fix is unlikely to happen in the near future. There is no other alternate library to read HDF5 files, including FAST5 files [3, 4].

Using a process pool

Multiple threads in a single process share the same address space and thus the lock in the HDF5 library affects multiple threads. Multiple threads are typically used to run sub-tasks in parallel while conveniently sharing data amongst the threads. In contrast, multiple processes have their own independent address spaces and are typically used to run isolated tasks in parallel. The presence of independent address spaces in multiple processes can be exploited to circumvent the lock in the HDF5 library.

A multi-process based solution is elaborated in **Extended Data Fig.1f**. Multi-threads in the single parent-process are used for data processing and multiple child-processes for I/O. The parent-process performs data processing using multiple threads in parallel. Each child-process has its own instance of the HDF5 library, as a consequence of independent address spaces. Moreover, each child-process has only a single thread that requests I/O. Thus, a single instance of the HDF5 library gets only one request at a time. In effect, there are multiple instances of the HDF5 library that can submit multiple I/O requests in parallel to the operating system (as opposed to the situation in multi-threaded HDF5 case), thus benefiting from the high throughput offered by RAID configurations.

Formally, if there are *K* processes and if the disk controller can serve *K* requests in parallel, the total time spent on I/O operations will be $T' = t \times \frac{n}{K}$.

Multiple processes are spawned at the beginning of the program using the *fork* system call. These forked child-processes form a pool of processes that exist until the lifetime of the parent-process, solely performing I/O of FAST5 files. The data processing can be performed by multiple threads spawned by the parent-process as usual. The parent-process, when it requires to load signal data of *N* reads (FAST5 accesses), first splits the list of reads into *K* parts where *K* is the number of child-processes. Then, each part is assigned to a child-process, which performs the assigned FAST5 accesses. When the data is loaded, the child-processes send this data to the parent-process.

Note 1: A fork-join model for multi-processes (as could be done for multi-threading) is unsuitable to be used instead of the process pool model presented above. Firstly, creating a process can be very expensive and could easily become the biggest bottleneck than the file reading itself. Secondly, forking in the middle of a program could double the memory usage and is usually problematic, and should be avoided where possible.

Note 2: It is important to note that *processes* in an operating system are meant for isolation whereas *threads* are for sharing data. Inter-process communication requires system calls, while inter-thread communication involves sharing the same memory space. Further, spawning multiple processes is expensive and is not lightweight (unlike threads). Thus, using processes as a replacement to threads makes the code relatively complicated.

In summary, a process pool approach, whilst technically viable, requires complicated re-engineering for every piece of software and is not a geralisable, long-term solution.

Naive Approaches of Multi-processing

Instead of using a process pool solely for FAST5 I/O and multi-threads for parallel data processing, developers may use multi-processes for both the I/O operations and parallel data processing. This would be easier than implementing a pool of processes, however, this is only suitable for perfectly parallel cases. This is the method we use in *slow5tools* for fast conversion from FAST5 to SLOW5. If the application needs to share data among multiple processing units, processes are unsuitable due to the complexity that arises when performing inter-process communication.

Alternatively, the developer may let users manually split data and launch multiple processes. Unfortunately, this method exerts additional burden on the user, i.e., custom scripts must be written for data splitting, launching data processing and concatenating the result. Moreover, this is only suitable for perfectly parallel applications where data can be easily split. Also, an expensive HPC system with dozens of cores is superfluous as the user could use a cluster of low cost networked computers [5].

ALTERNATIVES TO FAST5 FORMAT

Given the limitations to the FAST5 format highlighted above and the complexity of the 'workaround' solutions, we propose that a new file format is required. In the current article, we present 'SLOW5' format as our preferred solution. However other possible formats have been considered. These are discussed below.

Storing nanopore signal data in CRAM format

CRAM is a compressed columnar file format for storing biological sequences aligned to a reference sequence. CRAM was designed to be an efficient reference-based alternative to the SAM/BAM format. CRAM does not impose any rules about what data should or should not be preserved and CRAM has therefore been considered as a possible format for storing ONT signal data (https://github.com/EGA-archive/ont2cram). This seems appealing because CRAM is already an established format (for storing alignments) and is designed for efficient parallel access. However, implementation of efficient nanopore signal data storage faces several major technical hurdles:

1. CRAM is a genome-aligned format and therefore not ideal for storing data without a reference genome. This applies to a large fraction (if not the majority) of nanopore data. Additionally, raw data cannot be directly written in CRAM format since this first requires base-calling and alignment to a reference genome.

2. Within CRAM format, there is no way to efficiently access a particular record, given a read ID. CRAM indexing allows efficient queries by alignment coordinate, but not by read ID.

3. Data duplication at secondary/supplementary alignments would most likely cause very large file sizes for large Eukaryotic genome samples stored in a hypothetical CRAM format.

4. There is no way to store double precision floating point data in CRAM format. This data is required for lossless storage of nanopore data, so lossless data conversion would not be possible with *ont2cram*.

On the basis of these issues, we do not consider CRAM format to be a viable solution for storage of nanopore signal data. With no formal format specification and just a single pre-release version in 2019, it appears that the *ont2cram* project has since been abandoned.

Storing nanopore signals in a generic database format

Another proposed solution would be to retain FAST5 as the native format for ONT data generation and storage, whilst temporarily moving signal data into a database format during analysis. This seems appealing because it would take advantage of existing database formats like SQL or MongoDB. Just like HDF5, these formats can store any data in any structure, including nanopore signal data. However, a database of nanopore signal data would have a large storage footprint, be poorly compressible, highly coupled with the specific database management system and therefore not portable, and would fail to take advantage of the principles of temporal/spatial locality of data access that make domain-specific file formats highly efficient. Indeed, a generic database format is a potential solution for any data storage/access problem in computer science, but is rarely the most efficient solution. Domain-specific file formats (e.g. MP3, JPEG, PDF, DOCX, SAM, SLOW5), rather than generic database formats, are the norm in modern computer science.

REFERENCES

[1] Simpson, J. T. et al. Detecting DNA cytosine methylation using nanopore sequencing. *Nat. Methods* **14**, 407–410 (2017).

[2] hdfgroup.org, “Questions about thread-safety and concurrent access,” Accessed: Jul 30, 2020. [Online]. Available: https://portal.hdfgroup.org/display/knowledge/questions+about+thread-safety+and+concurrent+access

[3] C. Rossant, “Moving away from HDF5,” 2016, Accessed: Jul 28, 2020. [Online]. Available: https://cyrille.rossant.net/moving-away-hdf5/

[4] C. Rossant, “Should you use HDF5?” 2016, Accessed: Jul 28, 2020. [Online]. Available: https://cyrille.rossant.net/should-you-use-hdf5/

[5] R. P. Mohanty, H. Gamaarachchi, A. Lambert, and S. Parameswaran, “SWARAM: Portable Energy and Cost Efficient Embedded System for Genomic Processing,” *ACM Transactions on Embedded Computing Systems (TECS)* **18**, 1–24 (2019)

[6] M. H-Y. Fritz, R. Leinonen, G. Cochrane, and Ewan Birney, “Efficient storage of high throughput DNA sequencing data using reference-based compression”, *Genome Res.* **21**, 734–740 (2011).

Supplementary Note 3. SLOW5 specifications (version 0.2.0)

PREAMBLE

SLOW5 is a new file format encoding signal data from nanopore sequencing. SLOW5 was developed to overcome inherent limitations in the existing FAST5 (HDF5) data format that prevent efficient parallel analysis and cause many headaches for developers.

SLOW5 refers to two file formats, namely SLOW5 ASCII and SLOW5 binary (called BLOW5). The extension for SLOW5 ASCII is *.slow5* and for BLOW5 it is *.blow5*. For efficient data access and to minimise disk space, users are expected to use BLOW5. SLOW5 ASCII is the human readable format and should only be used to view the content.

Random access to either SLOW5 ASCII or BLOW5 is supported using a binary index file. This is a separate file in the same directory as the SLOW5 ASCII or BLOW5 file. For SLOW5 ASCII, the index takes the extension *.slow5.idx* and for BLOW5 the index takes *.blow5.idx*.

A SLOW5 file contains a header followed by the sequencing data. In datasets from Oxford Nanopore Technologies (ONT), the *run_id* is a unique identifier that distinguishes a sequencing run. We will refer to a sequencing run and its data as a *read group*. A SLOW5 file can store multiple read groups in a single file, allowing data from multiple sequencing runs to be stored in a single SLOW5 file, whilst retaining their individual metadata.

Full specifications for current and previous versions of SLOW5 are available at: https://hasindu2008.github.io/slow5specs/

SLOW5 ASCII

A SLOW5 ASCII file is a plain text file that uses the American Standard Code for Information Interchange (ASCII) encoding (locale: C/POSIX, code set: US-ASCII). The file extension is *.slow5.*

An example structure of a SLOW5 ASCII file with a single read group is provided in **Table 1.** An example structure of a SLOW5 ASCII with multiple read groups - i.e., multiple sequencing runs - is provided in **Table 2**. The column/row borders and cell colours are added to increase the readability. The actual format uses tabs ('\t') and newlines ('\n') as delimiters (**IMPORTANT**: '\r' or "\r\n" are not allowed). The first set of lines is the SLOW5 header. The header lines start with '#' or '@'. The remainder of the file encodes nanopore signal data in one read per line.

Table 1: Example of a SLOW5 ASCII file with a single read group.

Blue = global header. Yellow = data header. White = data records.

#slow5_version	1.0.0							
#num_read_groups	1							
@asic_id	0004A30B00232BEC							
@exp_start_time	2020-01-01T00:00:00Z							
@flow_cell_id	FAH00000							
@run_id	855cdb							
…	…							
#char*	uint32_t	double	double	double	double	uint64_t	int16_t*	...
#read_id	read_group	digitisation	offset	range	sampling_rate	len_raw_signal	raw_signal	...
read0	0	8192	6	1467.6	4000	123456	498,492,...	...
read1	0	8192	5	1467.6	4000	2000	491,491,...	...
…	…	…	…	…	…	…	…	...
readN	0	8192	3	1467.6	4000	3000	400,400,...	...

Table 2. Example of a SLOW5 ASCII file with multiple read groups.

Blue = global header. Yellow = data header. White = data records.

#slow5_version	1.0.0							
#num_read_groups	3							
@asic_id	0004A30B00232BEC		1004A30B00232BEC			2004A30B00232BEC		
@exp_start_time	2020-01-01T00:00:00Z		2020-01-01T00:00:00Z			2020-01-01T00:00:00Z		
@flow_cell_id	FAH00000		FAH00001			FAH00002		
@run_id	855cdb		855cd1			855cdc		
…	…		…			…		
#char*	uint32_t	double	double	double	double	uint64_t	int16_t*	...
#read_id	read_group	digitisation	offset	range	sampling_rate	len_raw_signal	raw_signal	...
read-0	1	8192	6	1467.6	4000	4000	498,492,...	...
read-1	0	8192	5	1467.6	4000	2000	491,491,...	...
…	…	…	…	…	…	…	…	...
read-N	2	8192	3	1467.6	4000	3000	400,400,...	...

SLOW5 Header

The SLOW5 header stores metadata regarding the experiment. Header lines start with either '#' or '@'. The header contains two parts: the **global header** (blue fields in tables above) and the **data header** (yellow fields in tables above).

Global header

The lines starting with '#' form the global header (blue fields above).

The header lines are as follows:

1. The first line of a SLOW5 ASCII file is a key-value pair that specifies the SLOW5 version. The key is separated from the value using a tab '\t'.
2. The second line specifies the number of read groups in the file. Observe that in the single read group file example (**Table 1**), the value for *num_read_groups* is set to 1. In the second example with three read groups (**Table 2**) the value is set to 3.
3. The last line of the header is always the field names for the subsequent per-read records.
4. The second last line of the header specifies the data types of each field for the subsequent per-read records (i.e., for the fields named in the last line of the header). Further information about the fields is provided in the **SLOW5 Data** section below.

Data header

The header lines that start with '@' form the **data header** (yellow fields above). These header lines contain ONT data attributes that are shared across multiple reads in a sequencing run (read group). For instance, the *run_id* and the *flow_cell_id* are common to all the reads in the read group and are therefore stored in the data header (**Table 1**). These data header lines should always lie after the first two mandatory global header lines and before the last two mandatory global header lines, as illustrated in **Tables 1** & **2**.

For a SLOW5 file containing a single *run_id*, data header lines are key-value pairs delimited by a tab '\t' (**Table 1**). When there are multiple *run_ids* present, the key is followed by a series of values delimited by tabs '\t' (**Table 2**). The first value is for the read group 0, the second value is for the read group 1, the third value is for the read group 2 and so on.

If any attribute value is missing from a given read group a "." is used.

As indicated by the '…' in **Table 1** & **2** after the *run_id* row, many other data header lines may exist, encoding many attributes associated with a given nanopore sequencing experiment.

The dataset headers are sorted in ascending order based on the native byte values (US-ASCII in C/POSIX locale) of the key. Using sorting, rather than a fixed order, ensures the SLOW5 file format can easily accommodate the addition or removal of attributes in the future. A list of possible data header attributes (not an exhaustive list) is provided in **Table 3** below (note: we did not develop FAST5 files; many of the definitions are based on information in [1]).

Table 3. Common SLOW5 header attributes.

Data header attribute key	Description	Example value
asic_id	Application Specific Integrated Circuit identifier (ASIC) of the flow cell (unique number of the chip), for tracking purposes.	213553007
asic_id_eeprom	The identifier of the ASIC's electrically erasable programmable read-only memory (EEPROM) of the flow cell.	5309577
asic_temp	The temperature in degrees celsius of the ASIC chip at the start of the sequencing run.	28.867193
asic_version	The version of ASIC being used.	IA02D
auto_update	Indicates whether auto-update in Minknow is enabled or not.	0
auto_update_source	The link to the Minknow update source.	https://mirror.oxfordnanoportal.com/software/MinKNOW/
barcoding_enabled	Indicates whether barcode demultiplexing is enabled during live basecalling.	0
bream_is_standard	Bream is one of the software for controlling sequencing.	0
configuration_version	The version of the configuration system in MinKNOW including the experiment scripts.	4.0.13
device_id	The serial ID of the MinION or device position for GridION/PromethION. Device position on GridION/PromethION refers to the ID of the bay (slot where the flow-cell is put) on the device.	X2
device_type	The device type (currently MinION, PromethION or GridION).	gridion
distribution_status	Stable vs dev/alpha/beta status.	stable
distribution_version	MinKNOW version.	20.06.9
exp_script_name	The name of the experiment script run along with optional parameters passed to it, based on what kits are selected in MinKNOW for sequencing.	sequencing/sequencing_MIN106_DNA:FLO-MIN106:SQK-LSK109
exp_script_purpose	The 'purpose' of the experiment script. For example, whether the experiment was a real sequencing run or a simulation playback.	sequencing_run
exp_start_time	Start time of sequencing run.	2020-09-08T01:23:21Z
experiment_duration_set	Indicates the duration of the experiment selected when starting the sequencing run (assumed to be in minutes)	4320
experiment_type	Indicates the type of the experiment, for instance, genomic_dna or rna.	genomic_dna
flow_cell_id	Unique ID for the flow-cell, used by ONT to track flow-cell metrics and warranty.	FAN43349
flow_cell_product_code	The type of flowcell (product code of the flowcell and pore type). These will be different based on R9.4.1, R10.3, R9.5, PromethION, etc.	FLO-MIN106
guppy_version	Guppy version being used by MinKNOW.	4.0.11+f1071ce
heatsink_temp	The temperature (in degrees celsius) of the heat sink on the ASIC at the start of the sequencing run.	33.996094

hostname	The hostname of the computer/machine doing the sequencing run.	GXB02243
installation_type	This is the MinKNOW install type.	nc
local_basecalling	Indicates if live base calling is enabled or not.	1
operating_system	The operating system and the version of the computer performing the sequencing run.	ubuntu 16.04
package	Relates to Bream [https://github.com/nanoporetech/minknow_lims_interface].	bream4
protocol_group_id	This is the unique ID given to the group of acquisition periods during a run, denoted by run_id. Multiple acquisition periods can occur during a single "run", depending on the protocol.	GLFN180082
protocol_run_id	This is a unique identifier for the experiment GROUP (just in case the name given by the user is not unique). This is the same for each run of the same experimental group.	f2c69573-5fef-43b8-8d81-9cb20634aa7c
protocol_start_time	The start time of the data acquisition periods for a protocol_group_id. Appeared in FAST5 2.3.	2021-08-26T15:34:52.186021+10:00
protocols_version	Allows MinKNOW to track various protocols for barcoding, kits, etc.	6.0.7
run_id	The unique run ID which will be different for each run (data acquisition period), even in the same experiment group. Whenever MINKNOW starts an experiment script for data acquisition, a new run_id is generated.	07770780274b0e3703f00d969291b1a37a5a6be1
sample_frequency	Typically the same as the sampling_frequency in the channel_id group.	4000
sample_id	Sample ID is the name given by the user for the sample.	NA12878
sequencing_kit	The sequencing kit used, for instance, sqk-lsk109 or sqk-rna002. [https://store.nanoporetech.com/sample-prep.html]	sqk-lsk109
usb_config	Various information about the connection between the flow-cell and the computer.	GridX5_fx3_1.1.3_ONT#MinION_fpga_1.1.1#bulk#Auto
version	MinKNOW version.	4.0.3

NOTE: Many of the attributes in **Table 3** are not used in a typical signal analysis experiment and many are also inconsistent between various FAST5 versions. Although they are unlikely to be used, these attributes are retained by default when converting from FAST5 to SLOW5 format (i.e. conversion is lossless by default). Note that the above list is not an exhaustive list. For instance, FAST5 files generated on the PromethION have additional attributes such as *hublett_board_id* and *satellite_firmware_version*.

SLOW5 Data

After the SLOW5 header, the actual data is encoded (white fields in **Tables 1** & **2**, above). Each line contains information about a single read and we refer to this as a record. Each record is made up of several fields that are tab delimited.

As mentioned earlier, the last header line specifies the name of each field. There are two types of fields:

- Primary fields are mandatory and arranged in a strict order. There are 8 primary fields, which are exemplified in **Table 1** & **2** (from the *read_id* field to *raw_signal* field)
- Auxiliary fields are optional and arranged in no strict order. There can be 0 or more auxiliary fields and these are denoted by the '…' after the *raw_signal* field in **Table 1** & **2**.

The second last header line specifies the data type of each primary & auxiliary field. For the primary fields the data types are always the same, whereas the auxiliary field types depend on the fields themselves. The supported data types in SLOW5 are:

- 8-bit, 16-bit, 32-bit and 64-bit signed integers (*int8_t*, *int16_t*, *int32_t*, *int64_t*) and corresponding 1D arrays (*int8_t**, *int16_t**, *int32_t**, *int64_t**)
- 8-bit, 16-bit, 32-bit and 64-bit unsigned integers (*uint8_t, uint16_t, uint32_t, uint64_t*) and corresponding 1D arrays (*uint8_t*, uint16_t*, uint32_t*, uint64_t**)
- IEEE 754 32-bit and 64-bit precision floating point (*float, double*) and corresponding 1D arrays (*float**, *double**)
- ASCII characters (*char*) and ASCII strings (*char*)*. Note: Tabs ('\t' and newline characters '\n' are not allowed in either)
- 8-bit enumeration type (*enum*) that consists of integral constants. Enumerations must be declared with the *enum* keyword followed by the comma-separated integral constants inside curly braces. eg: *enum{const1,const2,const3}.* Note that the integral-constant names are restricted to alphanumeric characters plus underscores, similar to that in the C programming language. The values for the integral-constant are assigned based on the order they are defined, for instance, *const1 = 0, const2* = *1* and *const3* = *2* in the above example. Note that enum in SLOW5 is restricted to 8-bit.

Primary fields

The 8 primary data fields in SLOW5 format are summarised in **Table 4** below. These fields are mandatory and must be arranged in the order that they appear in **Table 4**.

Table 4. Primary data fields in SLOW5 format.

Field name	Data type	Description	Example value
read_id	char*	A unique identifier for the read. This is a Universally unique identifier (UUID) version 4, and should be unique for any read from any device.	00592138-f120-4ab5-9916-c5567adb8e29
read_group	uint32_t	Read group identifier. More information in the subsequent text.	0
digitisation	double	The digitisation is the number of quantisation levels in the Analog to Digital Converter (ADC). That is, if the ADC is 12 bit, digitisation is 4096 (2^{12}).	8192.0
offset	double	The ADC offset error. This value is added when converting the signal to pico ampere.	10.0
range	double	The full scale measurement range in pico amperes.	1441.389892578125
sampling_rate	double	Sampling frequency of the ADC, i.e., the number of data points collected per second.	4000
len_raw_signal	uint64_t	The number of samples in the raw signal (length of the raw_signal vector below).	59676
raw_signal	int16_t*	The raw signal which are the direct acquisition values from the ADC and are comma separated.	1039,588,588,593,586….

Of the 8 primary fields, *read_group* is the only field that does not appear in ONT's FAST5 format but has been introduced in SLOW5. *read_group* identifiers allow reads from multiple sequencing runs to be stored in the same file. *read_group* is essentially an index (0-based index) that specifies where the data header values for a given read are to be found in the data header. For instance, in **Table 2**, *read0* has the *read_group* 1 which means that the second value of the three values for each header attribute contains information for that particular sequencing run (e.g. out of the three values for the *flow_cell_id* key, second one is FAH00001).

In the SLOW5 header, the *num_read_groups* specify how many read groups are present. For instance, in **Table 2**, there are 3 samples in the file and thus *num_read_groups* is equal to 3. Note that the following should always be true: 0 <= *read_group* < *num_read_groups*. *read_group* is always 0 for a single sample file (as it is in **Table 1**).

Datasets are separated into multiple read groups based on the *run_id* (which is a unique string for a sequencing run specified in the data header). The indexing order of the read groups (*read_group)* is determined by the order the FAST5 files are parsed during FAST5 to SLOW5 conversion. This *read_group is* an internal index used for enumerating. This index allows more efficient enumeration (less computation and saves disk space) than performing string comparisons if *run_id* string was stored in the data record for every read instead.

Primary fields contain all the information required for a typical nanopore signal-level analysis. The raw signal can be easily converted to pico-ampere using the following equation:

```
signal_in_pico_ampere = (raw_signal + offset) * range / digitisation
```

Auxiliary fields

SLOW5 files may contain 0 or more auxiliary data fields, some common examples of which are provided in **Table 5** below. These fields are optional and not bound by any strict order.

Table 5. Common auxiliary data fields in SLOW5 format.

Field name	Data type	Description	Example value
channel_number	char*	The channel number. A flow cell has multiple channels allowing multiple DNA/RNA strands to be sequenced in parallel. For instance, a MinION flow cell has 512 channels and thus can sequence 512 strands in parallel.	504
median_before	double	The estimated median current level immediately preceding the read. In most cases this can be used as an estimate of the open pore level. The open-pore state is when there is no strand inside the pore.	238.78225708007812
read_number	int32_t	A unique number within each channel counted upwards from zero. Note that not all reads generated are "strand" reads, but only strand reads are written to the final fast5 file, so some read numbers may be absent.	17981
start_mux	uint8_t	The MUX setting for the channel when the read began. Each channel contains one or more wells. For instance, a MinION flow cell has 4 wells per channel. The wells within a channel are connected to a multiplexer (MUX), a switch that controls which of the four wells in the channel is controlled and read out for sequencing.	4
start_time	uint64_t	The start time of the read. The unit for *start_tim*e is 'number of signal samples', so *start_time* has to be divided by sampling rate (sampling_rate) to get the start time in seconds (i.e. the time since the run was started)	335845487

Auxiliary fields contain all per-read information from ONT FAST5 files that we do not consider primary data fields (i.e., attributes that are not commonly used in signal-level analysis). If a value for a particular auxiliary field is unavailable for a given read it is represented with a ".".

It is important to note that auxiliary fields can be in any order, meaning the user should not rely on their order and instead should enumerate based on the field names and data types specified in the header. Any future per-read attributes added to FAST5 by ONT will be included as auxiliary fields in SLOW5. If ONT drops any attribute from FAST5, it will also be dropped in SLOW5.

The auxiliary fields are separated from each other and from the primary fields by using a tab '\t' as a delimiter. The elements in a field of 1D array data type (except *char** strings) are delimited by commas. Strings are stored as a series of characters, as usual, and the null terminating character is not stored.

BLOW5

A SLOW5 binary file or a BLOW5 file is the binary counterpart to a SLOW5 ASCII file. The file extension is *.blow5*. In BLOW5 format all multi-byte numbers are stored in little-endian, regardless of the machine's endianness.

A BLOW5 file can be either uncompressed or compressed. At present, three separate compression/decompression schemes have been implemented in *slow5lib*, namely: (*i*) Z-Library (*zlib*; also referred to as *gzip* or *DEFLATE)*, which is an established library that is available by default on almost all systems; (*ii*) Zstandard (*zstd*), which is a recent, open source compression algorithm developed by Facebook; and (*iii*) StreamVByte (*svb), which is a* recent integer compression technique that uses Google's Group Varint approach). *Zlib* and *zstd* are used for compressing SLOW5 records (a record is the collection of all primary and auxiliary fields of a particular read), whereas *svb* is for compressing the raw signal field alone. Our implementation supports first compressing the raw signal using *svb* and then compressing the SLOW5 record (now with the raw signal *svb* compressed) using *zlib* or *zstd*, at the user's discretion. Each read is compressed/decompressed independently from one another by using an individual compression stream for each read. Thus, multiple reads can be accessed and decompressed in parallel using multiple threads.

The use of *zstd* on top of *svb* compression is equivalent to ONT's custom '*vbz*' scheme (https://github.com/nanoporetech/vbz_compression), which uses these two open source algorithms for FAST5 compression. We also note that *slow5lib* has been designed such that any other suitable compression scheme can be easily integrated if necessary, making it future proof.

BLOW5 Header

The fields of the BLOW5 header are displayed in **Table 6** below. Note that despite being shown in a table for clarity, the fields in a BLOW5 file are stored serially in the exact order as they are in **Table 6**, without any tabs or newlines to separate the fields. The byte offset in the file (first column) and the size of the field in bytes (second column) are used to locate a particular field within a BLOW5 file.

The first field, the magic number, is a 6 byte string "BLOW5\1" used as a signature to identify the file format. The next three fields are for storing the BLOW5 file version and the value here is the same as in the SLOW5 ASCII counterpart. The 5th field indicates if the BLOW5 records are compressed or not and the compression method used if compressed. The 6th field is the number of read groups in the file, which have the same value and meaning as in the SLOW5 ASCII counterpart (described above). From SLOW5 v0.2.0 onwards, 7th field indicates if a special compression has been applied for the raw signal and the method used for that. Finally, 49 bytes are reserved for future fields. These reserved bytes that are unused in this version must be initialised to zeros.

Offset 64 contains an integer field that indicates the size of the upcoming variable-sized field, the SLOW5 ASCII header. The next field is the SLOW5 ASCII header, which is the same as in a SLOW5 ASCII file, with the following exceptions:

1. The first line of the SLOW5 header specifying the #slow5_version is removed as this is already stored at the beginning of the BLOW5 header;
2. The second line of the SLOW5 header specifying the #num_read_groups is also removed as this is also stored at the beginning of the BLOW5 header;

Apart from these exceptions, the complete SLOW5 ASCII header is stored, including tabs, newlines and the starting characters "#" and "@". Note that all header data values will be converted to ASCII strings, despite the data type for the corresponding fields in FAST5 files.

Table 6. Structure of a BLOW5 file header.

Offset	size (bytes)	Description	data type	Value
00	6	Magic number	char[6]	"BLOW5\1"
06	1	Major version number	uint8_t	0 to 255
07	1	Minor version number	uint8_t	0 to 255
08	1	Patch version number	uint8_t	0 to 255
09	1	Record compression method	uint8_t	0 to 255 (0 for none, 1 for zlib, 2 for zstd)[7]
10	4	Number of read groups	uint32_t	0 to 2^{32}-1
14	1	Signal compression method (from v0.2.0 onwards)	uint8_t	0 to 255 (0 for none, 1 for svb-zd)[8]
14	50	Reserved for future	-	-
64	4	Size of the SLOW5 header (without null character)	uint32_t	0 to 2^{32}-1
68		The plain text header of the SLOW5 (null character not stored; #slow5_version and #num_read_groups are removed as they are already in the binary header)	char[]	

BLOW5 Data

The SLOW5 data records are serially stored in binary format with each record individually compressed using the record compression method specified in the header (data is not compressed if no compression is specified in the header, that is, if the record compression method is set to 0). From SLOW5 v.0.2.0 onwards, a special compression can be optionally applied to the raw signal field. If such special compression is applied and if so the compression method used is specified in the header (signal compression method). The record compression is still applied to the record (on top of the compressed signal now) if the record compression method in the header is set.

Note that each record is individually compressed to allow efficient parallel access to different records simultaneously.

IMPORTANT: Each BLOW5 record is preceded by the size of the upcoming BLOW5 record in bytes (the size of the compressed record if compressed), which is an 8-byte uint64_t type unsigned integer. Storing this size is useful for faster and easier indexing of a BLOW5. We will refer to this special field as "*len_blow5_rec*" from here onwards.

The fields in an uncompressed BLOW5 record are displayed in **Table 7** below.

[7] *none* means uncompressed binary. *zlib* stands for the z-library which is also referred to as gzip or DEFLATE. zstd stands for the z-standard. Note that more compressions can be added in future without changing the SLOW5 file version.
[8] *none* means uncompressed binary. *svb-zd* stands for StreamVByte [2] with zig-zag delta encoding. Note that more compressions can be added in future without changing the SLOW5 file version.

Table 7. Structure of a BLOW5 record.

Size (bytes)	Description	Data type
2	string length of the read ID (without null character)	uint16_t
<variable> - based on preceding value	read ID (null character not stored)	char*
4	read group	uint32_t
8	digitisation	double
8	offset	double
8	range	double
8	sampling_rate	double
8	len_raw_signal	uint64_t
<variable> - based on preceding value	raw_signal	int16_t*
	<auxiliary fields>	

The first field is a uint16_t integer that specifies the size of the upcoming *read_id* string. Then comes the eight primary data fields explained under the SLOW5 ASCII section (see above), but now stored in binary. Note that the *raw_signal* field, which was a comma separated list in SLOW5 ASCII, is now a series of int16_t integers (each 2 bytes in size) stored serially without commas. The size of the *raw_signal* field in bytes in **Table 7** is determined by the product of the *len_raw_signal* and the size of int16_t, which is 2.

The *raw_signal* field in a BLOW5 record is followed by the auxiliary fields, as described above. The fields are stored in the same order and datatypes as specified in the header.

Primitive data types (*int8_t*, uint8_t, *int16_t*, u*int16_t, int32_t*, uint32_t, *int64_t, uint64_t, float, double*, *char, enum*) are stored such that: *int8_t*, *uint8_t, char and enum* taking 1 byte; *int16_t* and u*int16_t* taking 2 bytes, *int32_t*, uint32_t and *float* taking 4 bytes; and, *int64_t, uint64_t and double* taking 8 bytes as shown in **Table 8** below. Any missing data field (represented by a '.' in SLOW5 ASCII) is represented in BLOW5 by using the value stated in column 3 in **Table 8**. This special value that represents a missing value cannot be used to represent the real value.

Auxiliary fields of 1D array data types are stored with the length of the 1D array (the number of elements in the 1D array, not the size in bytes) in the form of an 8 byte unsigned integer (uint64_t) preceding the actual data in the array. The elements in 1D arrays are stored sequentially without any delimiting commas. The size of the array field in bytes is determined by the product of the length of the 1D array and the size of the corresponding primitive data type. A missing array field including for strings ("." in SLOW5 ASCII) is represented by storing 0 as the length of the array.

Table 8. Primitive data types used in BLOW5 format.

Data type	size (bytes)	Missing value representation
int8_t	1	INT8_MAX = 2^7-1
int16_t	2	INT16_MAX = 2^15-1
int32_t	4	INT32_MAX = 2^31-1
int64_t	8	INT64_MAX = 2^63-1
uint8_t	1	UINT8_MAX = 2^8-1
uint16_t	2	UINT16_MAX = 2^16-1
uint32_t	4	UINT32_MAX = 2^32-1
uint64_t	8	UINT64_MAX = 2^64-1
float	4	generic NaN value returned by *nanf("")*
double	8	generic NaN value returned by *nan("")*
char	1	'\0'
enum	1	UINT8_MAX = 2^8-1

BLOW5 Footer

A BLOW file should always end with the end of file (EOF) marker "5WOLB". This is useful for detecting file truncation.

SLOW5 INDEX

A SLOW5 index is a binary file that contains an index to facilitate random access to a SLOW5 ASCII or BLOW5 file based on the *read_id*. The extension of an index for a SLOW5 ASCII file is *.slow5.idx* and for a BLOW5 file is *.blow5.idx*. A SLOW5 index always takes the same binary form as described below, irrespective of whether it is for a SLOW5 ASCII or BLOW5 file.

SLOW5 Index Header

Table 9. SLOW5 index header structure.

Offset	size (bytes)	Description	data type	Value
00	9	Magic number	char[9]	"SLOW5IDX\1"
09	1	Major version number	uint8_t	0-255
10	1	Minor version number	uint8_t	0-255
11	1	Patch version number	uint8_t	0-255
12	52	Reserved for future	-	-

Note: The SLOW5 index version is the same as that of the SLOW5 version in the corresponding SLOW5 file.

SLOW5 Index Data

The SLOW5 index data records start from offset 64 of the file. The index should have a single record for every record in the corresponding SLOW5/BLOW5 file. An individual SLOW5 index record takes the following form:

Table 10. SLOW5 index data structure.

Size (bytes)	Description	Data type
2	String length of the read ID (without null character)	uint16_t
<variable> - based on preceding value	Read ID (null character not stored)	char*
8	For ASCII SLOW5: byte offset in the SLOW5 ASCII file that corresponds to the beginning of the data record.<br><br>For BLOW5: byte offset in the BLOW5 file that corresponds to the beginning of the *len_blow5_rec* that precedes the data record.	uint64_t
8	For ASCII SLOW5: size of the SLOW5 ASCII data record in bytes.<br><br>For BLOW5: size of the BLOW5 data record in bytes (the size of the compressed record if compressed) + the size of the *len_blow5_rec* preceding the record (which is 8 as the datatype of *len_blow5_rec* is uint64_t).	uint64_t

SLOW5 Index Footer

A SLOW5 index file should always end with the end of file marker *"XDI5WOLS"*. This is useful in detecting file truncation.

RATIONALE BEHIND SLOW5 DESIGN DECISIONS

In this section we provide the rationale behind certain design decisions and why these were preferred over other potential solutions. Please note that some of the following discussions are pretty technical and not for the faint-hearted.

- Why does SLOW5 have two types of fields, primary and auxiliary?
  - Primary fields are the essential elements of signal-based analysis. These essential elements are provided as primary fields for easy and quick accessibility.
  - Auxiliary fields are very application-specific and not generally used in existing signal-based analyses. Keeping these fields separate prevents convoluting the primary fields. Also, auxiliary fields can be in any order and are optional. Therefore, the SLOW5 format will not break when ONT adds or removes a field, and users can optionally choose to discard the auxiliary fields during FAST5 to SLOW5 conversion, in order to reduce file size and complexity.

- Why is SLOW5 one big file opposed to a number of small files?
  - Modern file systems support bigger files. With disk sizes continually growing, this will be increasingly true in the future.
  - Random accesses would require repeated expensive file open and close operations if multiple files are used (the default number of maximally open files in Linux is typically 1024).
  - In the case of a user requiring to perform process-level parallelism on a per-file basis, they could use *slow5tools split* to quickly split the files.
  - When archiving, users tend to tar the files into a single ball anyway if there were multiple files. So why not just create a single file that can be directly archived?
  - Most bioinformatics users are familiar with working on a single large file for a given sample in FASTQ, BAM or VCF format, so we thought it would be good to follow this approach.

- Why does SLOW5 support multiple sequencing runs in the same file?
  - In nanopore sequencing experiments, it is quite common to run more than one flow cell on a given sample, or create a new run id when a flow cell is washed and reloaded during a run. Allowing data from multiple *run_ids* to be stored in a single SLOW5 file means developers do not have to deal with manually accepting multiple files when analysing data from more than one run. It is generally more convenient to have all the data in a single file.

- Why are empty fields in SLOW5 ASCII represented by "."?
  - SLOW5 ASCII is only for human readability and having a "." is easier to read than empty fields. This is also easier to parse when using tools like *awk, sed* and *cut*. Popular formats like SAM, BED and VCF use "." for empty fields, so we chose to stick to this convention.
  - If a single "." is to become a valid field value (unlikely) in FAST5 which is not the case at the moment, we would introduce a workaround such as using "\." or ".." in the future.

- Why is there a version number for the slow5 index?
  - To make it future proof.
  - In the future, we can provide alternate *btree* based indexing for memory efficiency if required.

- Why does BLOW5 use little endian storage?

    - All modern systems seem to use little endian. We are not aware of any big endian systems still in use.

- Why is a tab used as the delimiter in SLOW5 ASCII?
    - Tab separators are easy to parse with *awk, sed, cut* and other command line tools. This also mimics the convention used in SAM, VCF, BED and other genomics formats.

- Why are # and @ used in the SLOW5 header?
    - To distinguish the SLOW5 format related attributes (SLOW5 global header) from nanopore related attributes (SLOW5 data header) we use the two symbols # and @. Both of those characters are not supposed to be used in read identifiers and therefore there is no confusion with the data records.
    - We considered '##' for SLOW5 global header and a '#' for the SLOW5 data header but we decided against this because if ONT introduces an attribute name that starts with a '#', this would cause a lot of problems for SLOW5.

- Why is native byte order sort used for the attribute names in the SLOW5 data header?
    - There are many different data attributes and these are quite hard to keep track of because they differ between different FAST5 versions. Sorting these makes it easy for a human to quickly locate the information they are after.
    - To prevent adhoc ordering which would make it difficult to parse.

- Why doesn't SLOW5 support the analysis group in FAST5 files?
    - SLOW5 is meant for storing raw signal data. Storing analysis data (e.g., basecalled FASTQ records) would convolute the file format. We believe those post processing information should be a separate file, as is the case in other areas of bioinformatics where, for example, raw reads (FASTQ), alignments (BAM) and variants (VCF) are stored in separate files with specific formats.

- Why is a SLOW5 index always in binary and no ASCII version?
    - For fast loading and space saving.
    - The index is primarily read by a computer and not particularly useful to a human.

- Do any of the float/double fields in SLOW5 ASCII become lossy when they are converted to ASCII strings?
    - Yes, some of them do (for example recurrent decimals). However, this is not an issue when FAST5 is directly converted to BLOW5 as floats/doubles are directly stored in binary. SLOW5 ASCII is meant for viewing the binary counterpart BLOW5 by humans and not meant to be used for data archiving or processing. We recommend using the default conversion setup in *slow5tools f2s* that converts FAST5 files to zlib compressed BLOW5 files initially and the later use *slow5tools view*.
    - In ASCII we could have stored the float/double fields in hexadecimal to make lossless, but then this is not as readable to humans as a natural representation like x.y

- Do the values stored in the data header attributes become lossy as floats are also converted to string?
    - Currently all the data header attributes in SLOW5 are stored as ASCII strings in FAST5 as well. So at the moment there is no loss.

- What happened to the duration attribute in FAST5?
    - The duration attribute has a bad history. A few years ago this used to be the length of the signal in seconds and now this is used by ONT to represent the length of the signal in terms of number of samples. To avoid ambiguity we store the length of the signal in terms of the number of samples in the field *len_raw_signal* in SLOW5, which is equivalent to the value in the duration attribute in FAST5.
    - If ONT decides to make the duration in seconds again, we can add this as an auxiliary field for SLOW5 while keeping the *len_raw_signal* intact in SLOW5 as the length of the signal is essential in signal analysis.

- What if the end of file markers “5WLOB” or “*XDI5WOLS*” occur in the middle of a file?
    - This is possible to happen if the data by any chance matches this pattern in binary. However, this is not an issue as the end of the file marker in BLOW5 is used to detect file truncation. That is, we check if the end of the file marker is present only if the EOF has been reached.
    - The case that the data at the end of a truncation is translated to an end of marker is extremely rare.

- Why are certain fields such as “digitisation” that seem to be identical across all reads in a given sequencing run present in data records, opposed to being header data attributes?
    - These are essential values for converting the raw signal. So it is quite convenient to have them adjacent to the raw signal. Also in case something happens to the header, the records will still be usable.
    - In the future, this digitisation attribute may no longer be the same across reads (as ONT stores this redundantly for each read unlike the header data attributes which are symbolic links).

- Are options header lines that start with ‘#’ supported in SLOW5?
    - No. Optional lines would complicate parsing and can include complicated situations where different users starting to use a header of their own and later causing confusions. If the requirement comes, we will introduce this in a future version.

- What if the forbidden ‘\t’ and ‘\n’ in data header values and auxiliary fields ever should become a valid character in FAST5?
    - At the moment they are forbidden to keep the file format simple. If this ever happens, in future versions we will allow ‘\\t’ or something.

MISCELLANEOUS

SLOW5 versioning

While forward-compatibility cannot be ensured, backward compatibility will be maintained where possible.

Versioning follows the *major.minor.patch* approach.

- The *patch* version is incremented for backwards compatible bug fixes.
- The *minor* version is incremented for backward compatible newer features and functions.

- The *major* version is incremented when potentially backward incompatible changes are introduced.

There are two independent tracked versions:

1. slow5 file and slow5 index file version
2. *slow5lib*, *pyslow5* and *slow5tools* versions

The slow5 file and slow5 index file version is independent from the *slow5lib*, *pyslow5* and *slow5tools* version and is used for checking compatibility.

slow5lib, pyslow5 and slow5tools versions are independently patched while maintaining compatibility, and are version synced during any stable release.

REFERENCES

[1] Oxford Nanopore Technologies. Read .fast5 files from the instrument. *technical_documents* https://community.nanoporetech.com/technical_documents/data-analysis/v/datd_5000_v1_revn_22aug2016/read-fast5-files-from-th (2016).

[2] Lemire, Daniel, Nathan Kurz, and Christoph Rupp. "Stream VByte: Faster byte-oriented integer compression." Information Processing Letters 130 (2018): 1-6.
