# Supplementary material for: Fast nanopore sequencing data analysis with SLOW5
Source: Nat Biotechnol. 2022 Jan 3;40(7):1026–9. doi: 10.1038/s41587-021-01147-4 (PMC9287168; doi:10.1038/s41587-021-01147-4)
Supplement: Supplementary file 2 — Reporting Summary [file 41587_2021_1147_MOESM2_ESM.pdf]

nature research

Corresponding author(s): Dr Ira W Deveson

Last updated by author(s): 29 October 2021

Reporting Summary

Nature Research wishes to improve the reproducibility of the work that we publish. This form provides structure for consistency and transparency in reporting. For further information on Nature Research policies, see our Editorial Policies and the Editorial Policy Checklist.

Statistics

For all statistical analyses, confirm that the following items are present in the figure legend, table legend, main text, or Methods section.

n/a	Confirmed	
☒	☐	The exact sample size (*n*) for each experimental group/condition, given as a discrete number and unit of measurement
☒	☐	A statement on whether measurements were taken from distinct samples or whether the same sample was measured repeatedly
☒	☐	The statistical test(s) used AND whether they are one- or two-sided<br>*Only common tests should be described solely by name; describe more complex techniques in the Methods section.*
☒	☐	A description of all covariates tested
☒	☐	A description of any assumptions or corrections, such as tests of normality and adjustment for multiple comparisons
☒	☐	A full description of the statistical parameters including central tendency (e.g. means) or other basic estimates (e.g. regression coefficient) AND variation (e.g. standard deviation) or associated estimates of uncertainty (e.g. confidence intervals)
☒	☐	For null hypothesis testing, the test statistic (e.g. *F*, *t*, *r*) with confidence intervals, effect sizes, degrees of freedom and *P* value noted<br>*Give P values as exact values whenever suitable.*
☒	☐	For Bayesian analysis, information on the choice of priors and Markov chain Monte Carlo settings
☒	☐	For hierarchical and complex designs, identification of the appropriate level for tests and full reporting of outcomes
☒	☐	Estimates of effect sizes (e.g. Cohen's *d*, Pearson's *r*), indicating how they were calculated

Our web collection on statistics for biologists contains articles on many of the points above.

Software and code

Policy information about availability of computer code

Data collection

Raw-signal data for the benchmarking datasets described in Supplementary Table 1 were collected as multi-FAST5 files (zlib compression), using MinKNOW (distribution version 20.06.9, core version 4.0.3, configuration version 4.0.13). For benchmarking experiments where FAST5-vbz files were used, these were created using ONT's file compress_fast5 tool (version 4.0.0), which is part of the ont_fast5_api (https://github.com/nanoporetech/ont_fast5_api). FAST5 files were converted to SLOW5/BLOW5 files using slow5tools v0.3.0 built on top of slow5lib v0.3.0.

Data analysis

To perform computational benchmarking experiments at realistic workloads, we integrated slow5lib to f5c v0.2 CPU version (available: https://github.com/hasindu2008/f5c/tree/slow5-ioprof), which is a restructured version of Nanopolish that enables us to accurately measure the time for each individual component of a methylation calling job. FAST5 benchmarks were performed using the same version of f5c that uses HDF5 (1.10.4) built with the threadsafe option enabled (available: https://github.com/hasindu2008/f5c/tree/fastt-ioprof
). POSIX threads are used in f5c to perform multithreaded access to FAST5 and SLOW5. To obtain FASTQ files for methylation calling, Guppy 4.0.11 was used for base-calling under the dna_r9.4.1_450bps_hac_prom base-calling profile. To obtain the BAM file for methylation calling, the reads were mapped to the hg38 reference genome (with no alternate contigs) using minimap2 version 2.17-r941 (with -x map-ont -a --secondary=no options) and sorted using samtools v1.9.

SLOW5 format and all associated software are free and open source:
SLOW5 format specification documents can be accessed at: https://hasindu2008.github.io/slow5specs
Slow5lib/pyslow5 can be accessed at: https://hasindu2008.github.io/slow5lib/
Slow5tools can be accessed at: https://hasindu2008.github.io/slow5tools/
Custom branches of f5c used to measure internal operation times during benchmarking experiments are available at:
https://github.com/hasindu2008/f5c/tree/slow5-ioprof
https://github.com/hasindu2008/f5c/tree/fastt-ioprof

For manuscripts utilizing custom algorithms or software that are central to the research but not yet described in published literature, software must be made available to editors and reviewers. We strongly encourage code deposition in a community repository (e.g. GitHub). See the Nature Research guidelines for submitting code & software for further information.

Data

Policy information about availability of data

All manuscripts must include a data availability statement. This statement should provide the following information, where applicable:

- Accession codes, unique identifiers, or web links for publicly available datasets
- A list of figures that have associated raw data
- A description of any restrictions on data availability

Datasets used in benchmarking experiments are described in Supplementary Table 1 and are available on NCBI Sequence Read Archive at Bioproject PRJNA744329 with the following SRA accession numbers.
SRX11368473: ~30X human genome (NA12878) raw-signal data
SRX11368474: ~30X human genome (NA12878) alignments
SRX11368472: ~30X human genome (NA12878) basecalled sequences
SRX11368475: Downsampled human dataset (NA12878)
External datasets used in file-size comparisons are publicly available at various SRA accessions, as detailed in Supplementary Table 3.

Field-specific reporting

Please select the one below that is the best fit for your research. If you are not sure, read the appropriate sections before making your selection.

☒ Life sciences ☐ Behavioural & social sciences ☐ Ecological, evolutionary & environmental sciences

For a reference copy of the document with all sections, see nature.com/documents/nr-reporting-summary-flat.pdf

Life sciences study design

All studies must disclose on these points even when the disclosure is negative.

Sample size	n =1. Benchmarking experiments were performed with a single dataset comprising ONT sequencing reads from human genomic DNA. for different experiments, the full 30X dataset or a small, downsampled version of 500 million reads (see Supplementary Table 1). These datasets are typical of what would be encountered in ONT data analysis. A range of other datasets, each n=1, were used in file size comparisons, as detailed in Supplementary Table 3.<br>n=1 samples are sufficient for computational benchmarking experiments because these are deterministic by their nature, and not affected by biological/experimental variables.
Data exclusions	No data was excluded.
Replication	n=1. No biological conclusions are drawn and no statistical tests were performed. This is a computational benchmarking / proof-of-priniciple study, and replication is not relevant/necessary. n=1 replication is sufficient for computational benchmarking experiments because these are deterministic by their nature, and not affected by biological/experimental variables.
Randomization	No biological conclusions are drawn and no statistical tests were performed. This is a computational benchmarking / proof-of-priniciple study, and randomisation is not relevant/necessary.
Blinding	No biological conclusions are drawn and no statistical tests were performed. This is a computational benchmarking / proof-of-priniciple study, and blinding is not relevant/necessary.

Reporting for specific materials, systems and methods

We require information from authors about some types of materials, experimental systems and methods used in many studies. Here, indicate whether each material, system or method listed is relevant to your study. If you are not sure if a list item applies to your research, read the appropriate section before selecting a response.

Materials & experimental systems

n/a	Involved in the study
☒	☐ Antibodies
☒	☐ Eukaryotic cell lines
☒	☐ Palaeontology and archaeology
☒	☐ Animals and other organisms
☒	☐ Human research participants
☒	☐ Clinical data
☒	☐ Dual use research of concern

Methods

n/a	Involved in the study
☒	☐ ChIP-seq
☒	☐ Flow cytometry
☒	☐ MRI-based neuroimaging
